# Supplementary figures and images for: Development of photosynthetic carbon fixation model using multi-excitation wavelength fast repetition rate fluorometry in Lake Biwa
Source: PLoS One. 2021 Feb 2;16(2):e0238013. doi: 10.1371/journal.pone.0238013 (PMC7853527; doi:10.1371/journal.pone.0238013)

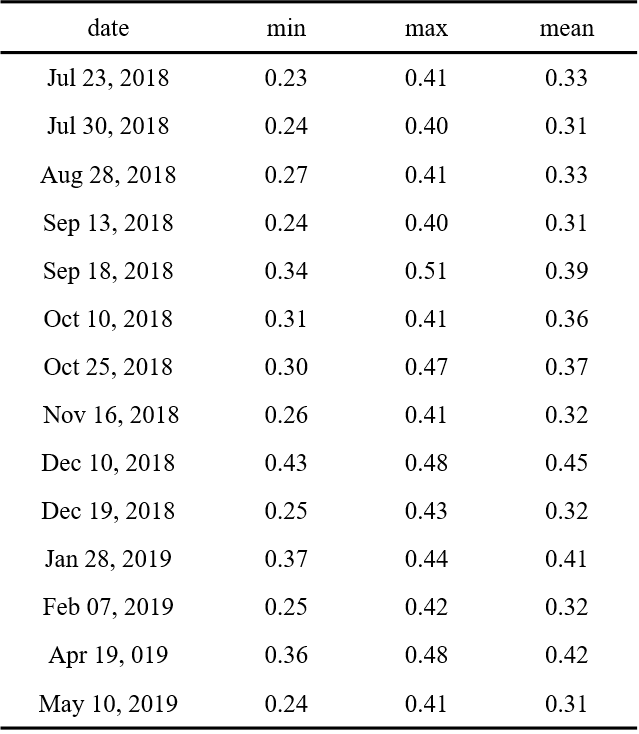


**S1. Table. Spectral correction factor (SCF) for *JVf* estimation in each sampling date.**

Supplement: S1 Table — (DOCX) [file pone.0238013.s001.docx]

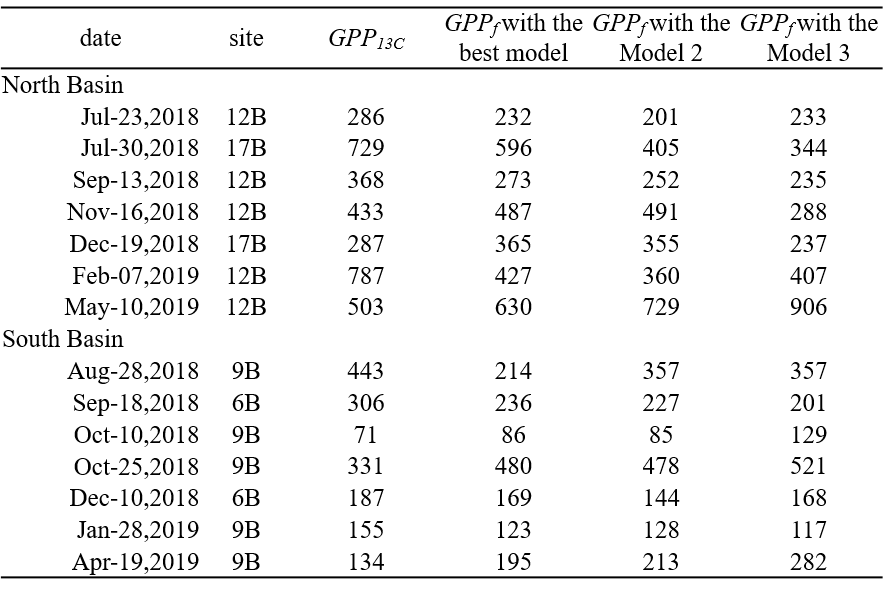


**S4. Table. GPP (g C m−2 d−1) estimated by 13C and FRRf with Фe,C models in each sampling date.**

Supplement: S4 Table — (DOCX) [file pone.0238013.s004.docx]

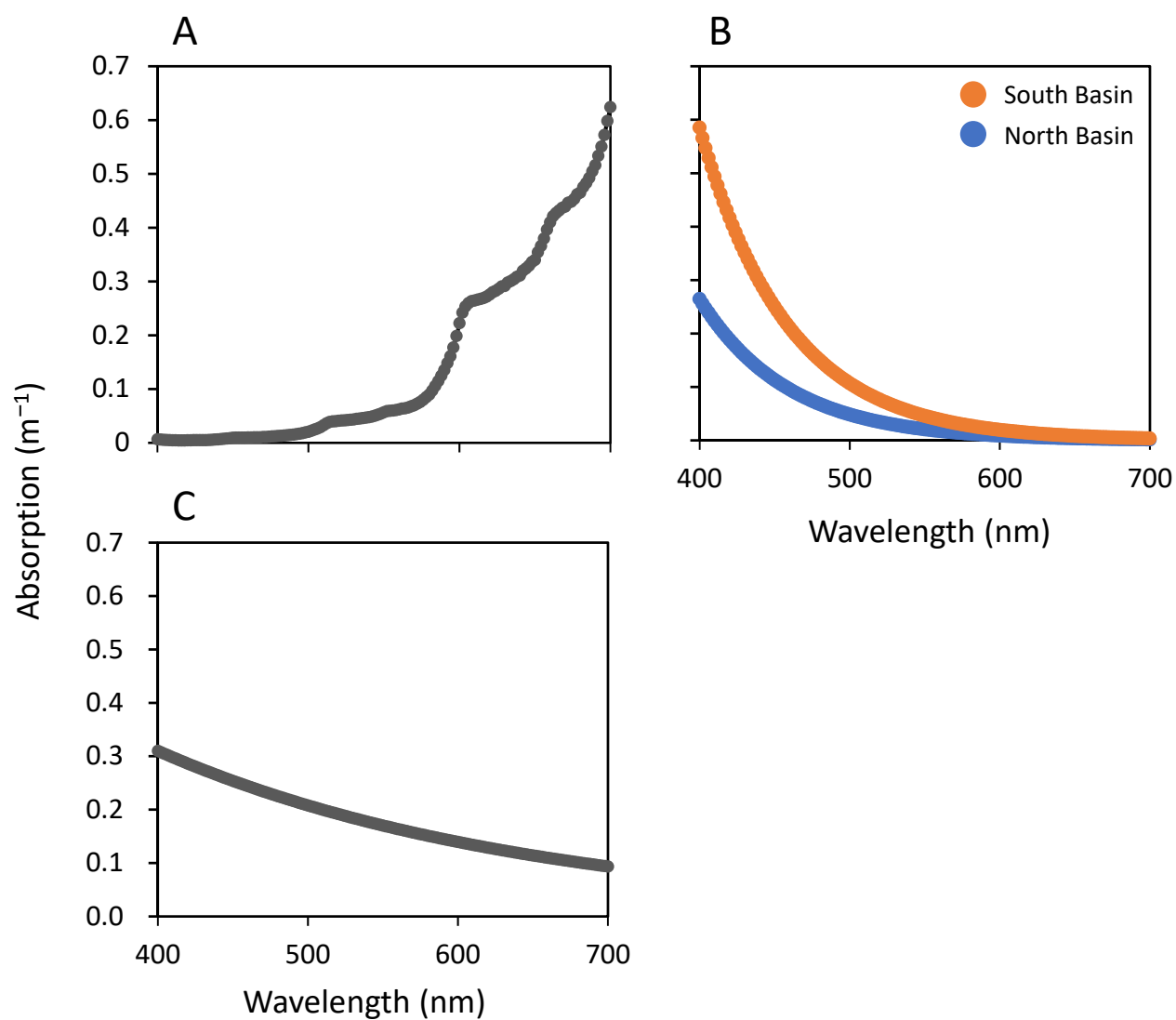

**S4 Appendix. Modeled absorption spectrum of (A) pure water, (B) CDOM and (C) non-algal particles.**

Supplement: S4 Appendix — Modelled absorption spectra of (A) pure water, (B) CDOM and (C) non-algal particles. (PDF) [file pone.0238013.s008.pdf]
