# Supplementary material for: Development of photosynthetic carbon fixation model using multi-excitation wavelength fast repetition rate fluorometry in Lake Biwa
Source: PLoS One. 2021 Feb 2;16(2):e0238013. doi: 10.1371/journal.pone.0238013 (PMC7853527; doi:10.1371/journal.pone.0238013)
Supplement: S1 Appendix — Spectral distribution of (A) excitation flash of FastOcean (B) light source of growth chamber. (PDF) [file pone.0238013.s005.pdf]

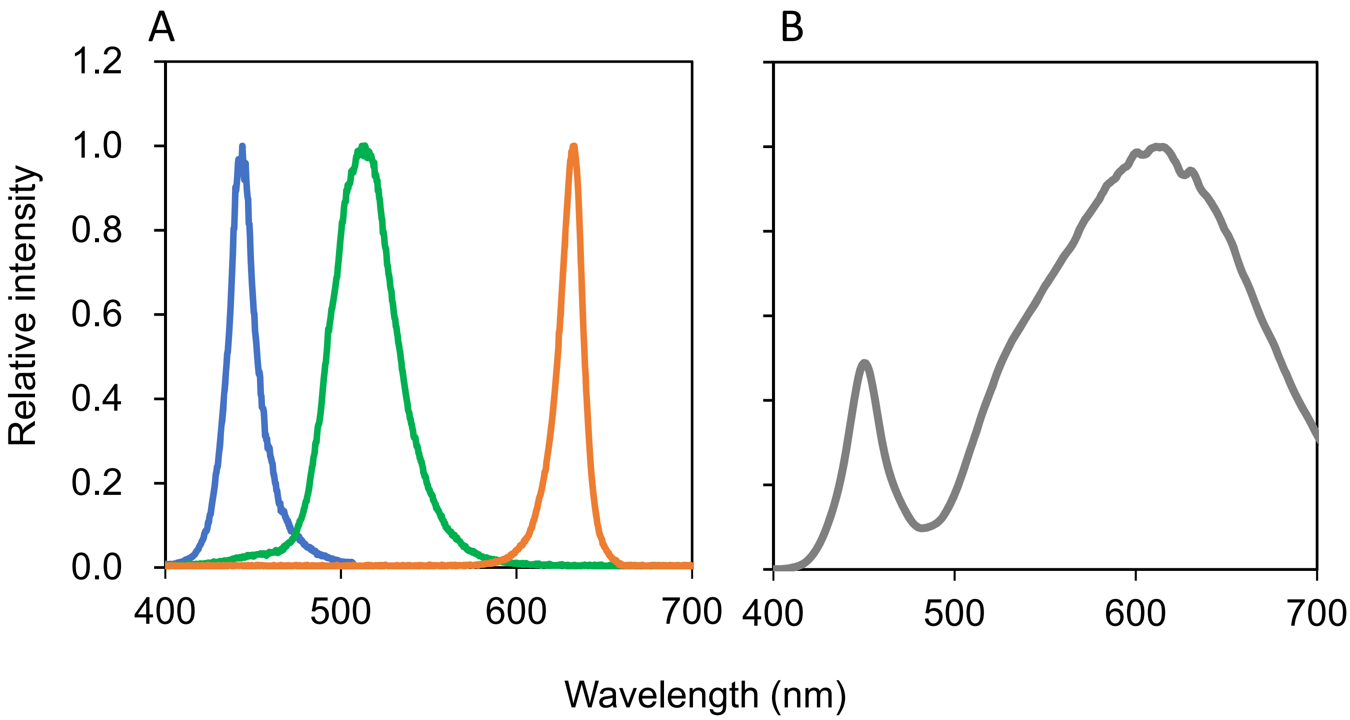

S1 Appendix. Spectral distribution of (A) excitation flash of FastOcean (B) light source of growth-chamber.
