# Supplementary material for: Development of photosynthetic carbon fixation model using multi-excitation wavelength fast repetition rate fluorometry in Lake Biwa
Source: PLoS One. 2021 Feb 2;16(2):e0238013. doi: 10.1371/journal.pone.0238013 (PMC7853527; doi:10.1371/journal.pone.0238013)
Supplement: S2 Appendix — The combination of three excitation wavelengths was used. For Jf, PAR intensity was corrected by white background light intensity of the Act2 system and SCF (see Materials and methods). The fitted curve is given by a two-parameter model [80]. (PDF) [file pone.0238013.s006.pdf]

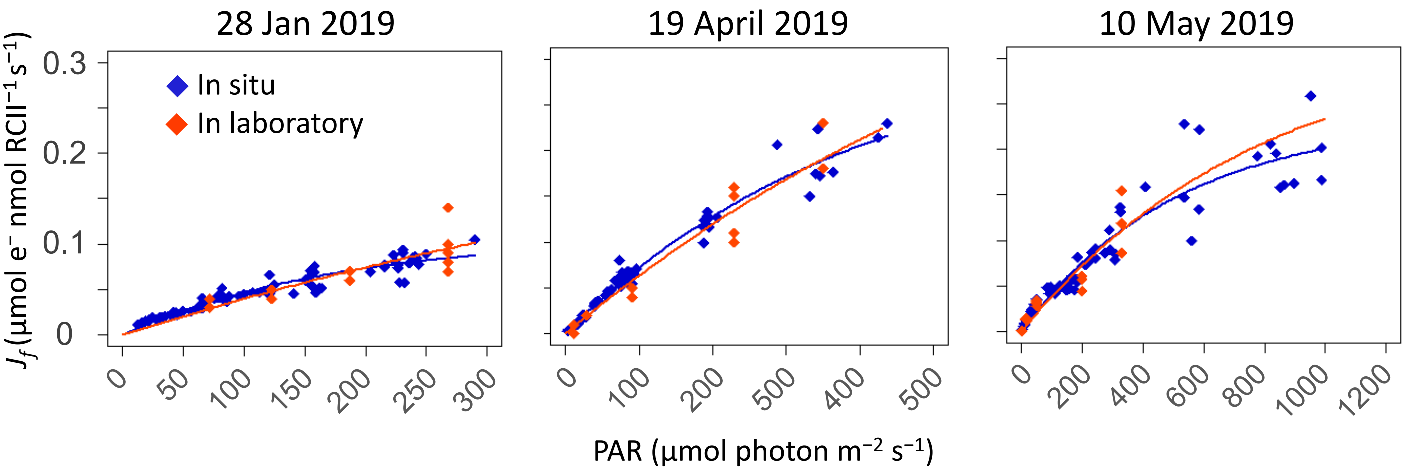

S2 Appendix. Scatter plots of J_f in *in situ* in morning, and in laboratory in afternoon (at the start of the incubation experiments). The combinations of three excitation wavelength was used. For J_f, PAR intensity was corrected by white background light intensity of Act2 system and SCF (see Materials and methods). The fitted curve is given by a two-parameter model [79].

.
