# Supplementary material for: Development of photosynthetic carbon fixation model using multi-excitation wavelength fast repetition rate fluorometry in Lake Biwa
Source: PLoS One. 2021 Feb 2;16(2):e0238013. doi: 10.1371/journal.pone.0238013 (PMC7853527; doi:10.1371/journal.pone.0238013)
Supplement: S3 Appendix — The spectra were calculated with Paavel’s model [86] for 30 and 40 μg L−1 in August, and Ylöstalo’s model [87] for the others. (PDF) [file pone.0238013.s007.pdf]

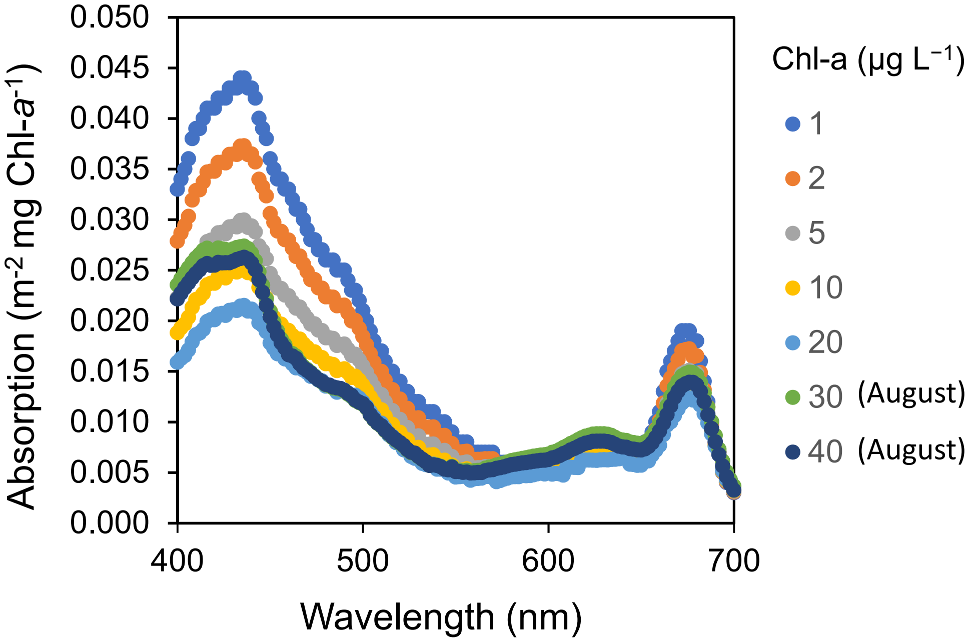

S3 Appendix. Modeled absorption spectrum for various Chl-*a* concentrations. The spectrum were calculated with Paavel's model [85] for 30 and 40 μg L^{-1} in August, and Ylöstalo's model [86] for others.
