# Supplementary material for: Development of photosynthetic carbon fixation model using multi-excitation wavelength fast repetition rate fluorometry in Lake Biwa
Source: PLoS One. 2021 Feb 2;16(2):e0238013. doi: 10.1371/journal.pone.0238013 (PMC7853527; doi:10.1371/journal.pone.0238013)
Supplement: S5 Appendix — Spectral distribution of incident sunlight at 10:00 in (A) April to September, (B) October to February and (C) at 4 PM in July in 2015. Each spectrum distribution was referenced to calculate the spectral correction on each sampling date as in Eq (7). (PDF) [file pone.0238013.s009.pdf]

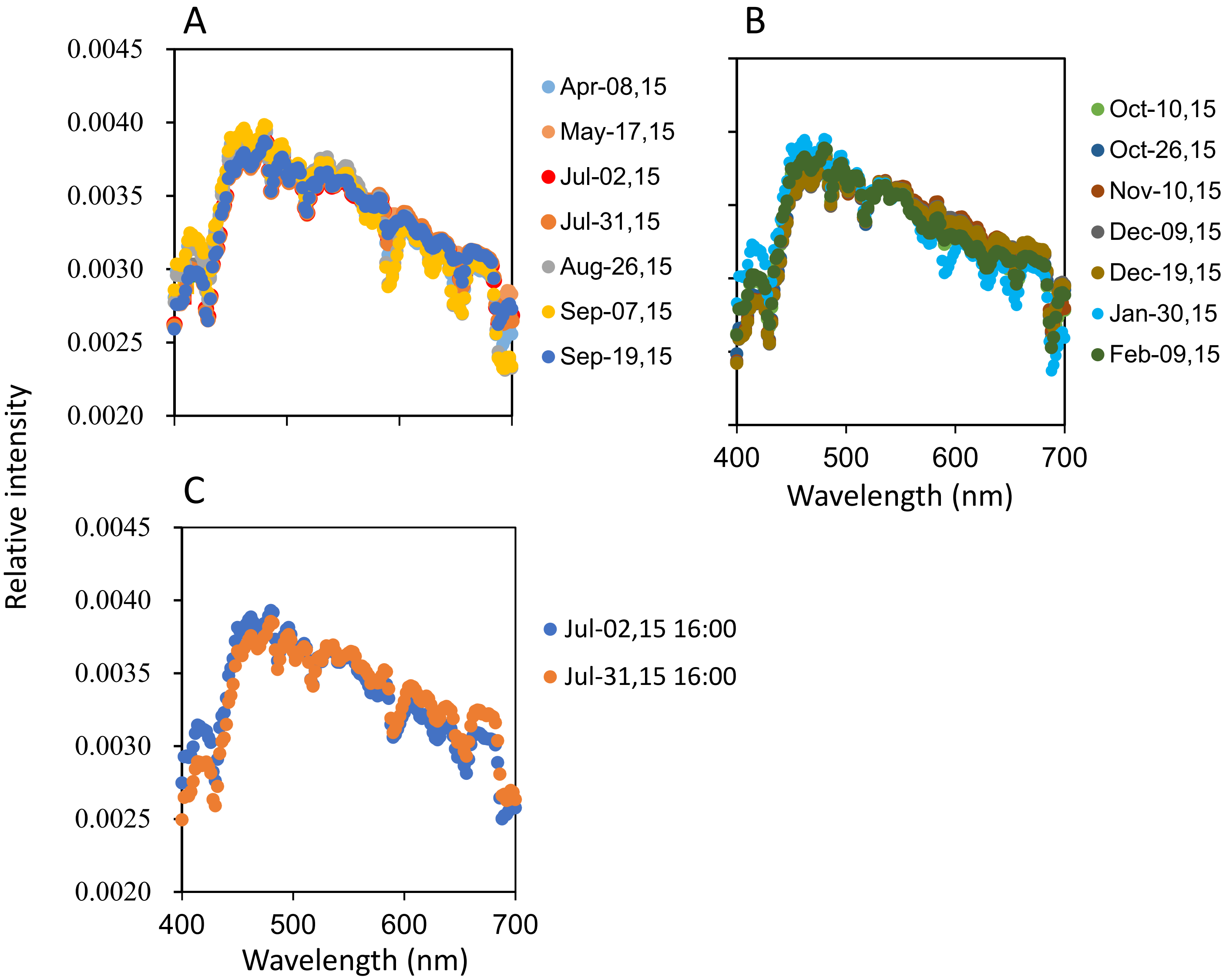

S5 Appendix. Spectral distribution of incident sunlight at 10:00 in (A) April to September, (B) October to February and (C) at 4 PM in July in 2015. Each spectrum distribution was referred to calculate the spectral correction in each sampling date as in equation (6).
