# Supplementary material for: Development of photosynthetic carbon fixation model using multi-excitation wavelength fast repetition rate fluorometry in Lake Biwa
Source: PLoS One. 2021 Feb 2;16(2):e0238013. doi: 10.1371/journal.pone.0238013 (PMC7853527; doi:10.1371/journal.pone.0238013)
Supplement: S6 Appendix — (PDF) [file pone.0238013.s010.pdf]

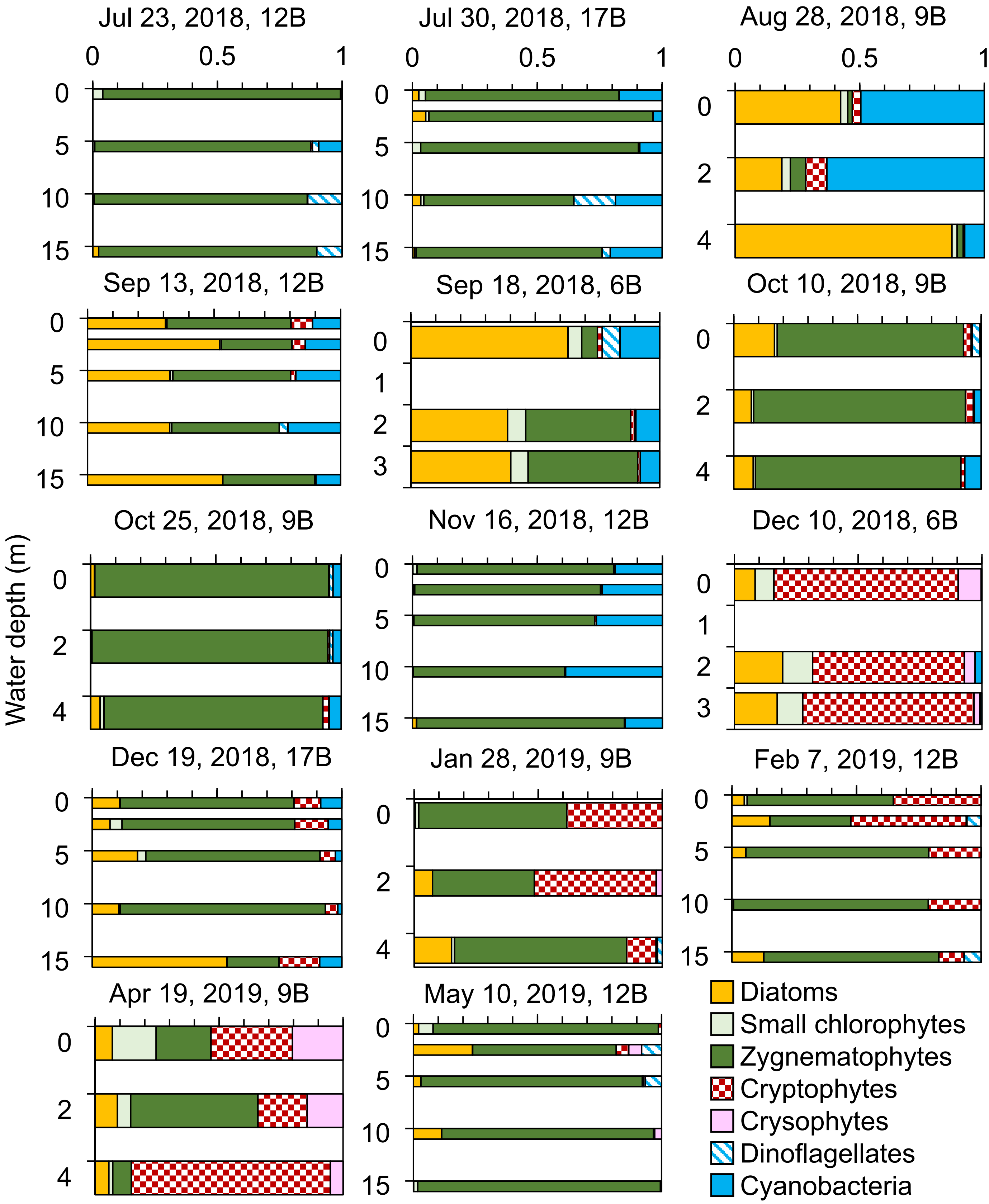

S6 Appendix. Relative contribution to total phytoplankton biomass by algal groups on each sampling date.
