# Supplementary material for: Development of photosynthetic carbon fixation model using multi-excitation wavelength fast repetition rate fluorometry in Lake Biwa
Source: PLoS One. 2021 Feb 2;16(2):e0238013. doi: 10.1371/journal.pone.0238013 (PMC7853527; doi:10.1371/journal.pone.0238013)
Supplement: Attachment — Submitted filename: PONE-D-20-24363_reviewer rmf.pdf [file pone.0238013.s011.pdf]

PLOS ONE

Development of photosynthetic carbon fixation model using multi-excitation wavelength fast repetition rate fluorometry in Lake Biwa

--Manuscript Draft--

**Manuscript Number:**	PONE-D-20-24363
**Article Type:**	Research Article
**Full Title:**	Development of photosynthetic carbon fixation model using multi-excitation wavelength fast repetition rate fluorometry in Lake Biwa
**Short Title:**	Carbon fixation model using multi-excitation wavelength FRRf in Lake Biwa
**Corresponding Author:**	Takehiro Kazama<br>National Institute for Environmental Studies<br>Otsu, Shiga JAPAN
**Keywords:**	environmental science; Field ecology; Fluorometry; Limnology; FRRf; Photosynthesis
**Abstract:**	Direct measurements of gross primary productivity (GPP) in the water column are essential, but can be spatially and temporally restrictive. Fast repetition rate fluorometry (FRRf) is a bio-optical technique based on chlorophyll *a* (Chl-*a*) fluorescence that can estimate the electron transport rate (ETR_{PSII}) at photosystem II (PSII) of phytoplankton in real time. However, derivation of phytoplankton GPP in carbon units from ETR_{PSII} remains challenging because the electron requirement for carbon fixation ($\Phi_{e,C}$) can vary depending on multiple factors. Also, the FRRf is still relatively novel, especially in freshwater ecosystems where phosphorus limitation and cyanobacterial blooms are common. The goal of the present study is to construct a robust $\Phi_{e,C}$ model for freshwater ecosystems using simultaneous measurements of ETR_{PSII} by FRRf with multi-excitation wavelengths coupled with traditional carbon fixation rate by the ^{13}C method. The study was conducted in oligotrophic and mesotrophic areas in Lake Biwa from July 2018 to May 2019. The combination of excitation light at 444, 512 and 633 nm correctly estimated ETR_{PSII} of cyanobacteria. The range of $\Phi_{e,C}$ in the phytoplankton community varied from 1.1 to 31.0 mol e^{-} mol C^{-1} during the study period. Generalized liner model showed the best model including 12 physicochemical and biological factors explained 67% of the variance in $\Phi_{e,C}$. Among all factors, water temperature was the most significant, while PAR intensity was not. The GPP values estimated by FRRf (GPP_f) with the best $\Phi_{e,C}$ model relative to ^{13}C (GPP_{13C}) varied 0.5–1.5. Further, GPP_f estimated with more parsimonious $\Phi_{e,C}$ models were also comparable to GPP_{13C}. This study quantifies the applicability of the *in situ* FRRf methodology, and supports continuous monitoring of GPP by FRRf in lakes with large spatio-temporal variability of environmental conditions and phytoplankton assemblages.
**Order of Authors:**	Takehiro Kazama
	Kazuhide Hayakawa
	Victor S. Kuwahara
	Koichi Shimotori
	Akio Imai
	Kazuhiro Komatsu
**Additional Information:**	
**Question**	**Response**
**Financial Disclosure**<br><br>Enter a financial disclosure statement that describes the sources of funding for the work included in this submission. Review	TK, KH, KS and AI were supported by the Collaborative Research Fund from Shiga Prefecture entitled "Study on water quality and lake-bottom environment for protection of the soundness of water environment" under the Japanese Grant for Regional Revitalization, and the Environment Research and Technology Development Fund (No. 5-1607) of the Ministry of the Environment,

Powered by Editorial Manager® and ProduXion Manager® from Aries Systems Corporation

the submission guidelines for detailed requirements. View published research articles from *PLOS ONE* for specific examples.<br><br>This statement is required for submission and **will appear in the published article** if the submission is accepted. Please make sure it is accurate.<br><br>**Unfunded studies**<br>Enter: *The author(s) received no specific funding for this work.*<br><br>**Funded studies**<br>Enter a statement with the following details:<br>• Initials of the authors who received each award<br>• Grant numbers awarded to each author<br>• The full name of each funder<br>• URL of each funder website<br>• Did the sponsors or funders play any role in the study design, data collection and analysis, decision to publish, or preparation of the manuscript?<br>• **NO** - Include this sentence at the end of your statement: *The funders had no role in study design, data collection and analysis, decision to publish, or preparation of the manuscript.*<br>• **YES** - Specify the role(s) played.<br><br>* typeset	Japan.https://www.kantei.go.jp/jp/singi/tiiki/tiikisaisei/souseikoufukin.html
**Competing Interests**<br><br>Use the instructions below to enter a competing interest statement for this submission. On behalf of all authors, disclose any competing interests that could be perceived to bias this work—acknowledging all financial support and any other relevant financial or non-financial competing interests.<br><br>This statement **will appear in the published article** if the submission is accepted. Please make sure it is accurate. View published research articles from *PLOS ONE* for specific examples.	The authors have declared that no competing interests exist.

Powered by Editorial Manager® and ProduXion Manager® from Aries Systems Corporation

**NO authors have competing interests**<br><br>Enter: *The authors have declared that no competing interests exist.*<br><br>**Authors with competing interests**<br><br>Enter competing interest details beginning with this statement:<br><br>*I have read the journal's policy and the authors of this manuscript have the following competing interests: [insert competing interests here]*<br><br>* typeset	
**Ethics Statement**<br><br>Enter an ethics statement for this submission. This statement is required if the study involved:<br><br>• Human participants<br>• Human specimens or tissue<br>• Vertebrate animals or cephalopods<br>• Vertebrate embryos or tissues<br>• Field research<br><br>Write "N/A" if the submission does not require an ethics statement.<br><br>General guidance is provided below. Consult the submission guidelines for detailed instructions. **Make sure that all information entered here is included in the Methods section of the manuscript.**	N/A

Powered by Editorial Manager® and ProduXion Manager® from Aries Systems Corporation

**Format for specific study types**<br><br>**Human Subject Research (involving human participants and/or tissue)**<br>• Give the name of the institutional review board or ethics committee that approved the study<br>• Include the approval number and/or a statement indicating approval of this research<br>• Indicate the form of consent obtained (written/oral) or the reason that consent was not obtained (e.g. the data were analyzed anonymously)<br><br>**Animal Research (involving vertebrate animals, embryos or tissues)**<br>• Provide the name of the Institutional Animal Care and Use Committee (IACUC) or other relevant ethics board that reviewed the study protocol, and indicate whether they approved this research or granted a formal waiver of ethical approval<br>• Include an approval number if one was obtained<br>• If the study involved *non-human primates*, add *additional details* about animal welfare and steps taken to ameliorate suffering<br>• If anesthesia, euthanasia, or any kind of animal sacrifice is part of the study, include briefly which substances and/or methods were applied<br><br>**Field Research**<br><br>Include the following details if this study involves the collection of plant, animal, or other materials from a natural setting:<br>• Field permit number<br>• Name of the institution or relevant body that granted permission	
**Data Availability**<br><br>Authors are required to make all data underlying the findings described fully available, without restriction, and from the time of publication. PLOS allows rare exceptions to address legal and ethical concerns. See the PLOS Data Policy and FAQ for detailed information.	Yes - all data are fully available without restriction

A Data Availability Statement describing where the data can be found is required at submission. Your answers to this question constitute the Data Availability Statement and **will be published in the article**, if accepted.<br><br>**Important:** Stating 'data available on request from the author' is not sufficient. If your data are only available upon request, select 'No' for the first question and explain your exceptional situation in the text box.<br><br>Do the authors confirm that all data underlying the findings described in their manuscript are fully available without restriction?	
**Describe where the data may be found in full sentences. If you are copying our sample text, replace any instances of XXX with the appropriate details.**<br><br>• If the data are **held or will be held in a public repository**, include URLs, accession numbers or DOIs. If this information will only be available after acceptance, indicate this by ticking the box below. For example: *All XXX files are available from the XXX database (accession number(s) XXX, XXX.)*.<br>• If the data are all contained **within the manuscript and/or Supporting Information files**, enter the following: *All relevant data are within the manuscript and its Supporting Information files.*<br>• If neither of these applies but you are able to provide **details of access elsewhere**, with or without limitations, please do so. For example:<br><br>*Data cannot be shared publicly because of [XXX]. Data are available from the XXX Institutional Data Access / Ethics Committee (contact via XXX) for researchers who meet the criteria for access to confidential data.*<br><br>*The data underlying the results presented in the study are available from (include the name of the third party*	All relevant data are within the manuscript and its Supporting Information files.

and contact information or URL).<br>• This text is appropriate if the data are owned by a third party and authors do not have permission to share the data.<br><br>* typeset	
Additional data availability information:	

Powered by Editorial Manager® and ProduXion Manager® from Aries Systems Corporation

Manuscript Click here to access/download;Manuscript;FRRfgpp_ver7.2.docx

Manuscript for submission to *PLOS ONE*

Development of photosynthetic carbon fixation model using multi-excitation wavelength fast repetition rate fluorometry in Lake Biwa

Last revision: 20/8/4

Takehiro Kazama[1,2*], Kazuhide Hayakawa[3], Victor S. Kuwahara[4], Koichi Shimotori[1,2], Akio Imai[1], Kazuhiro Komatsu[2]

1) Lake Biwa Branch Office, National Institute for Environmental Studies, Otsu, Shiga, Japan

2) Center for Regional Environmental Research, National Institute for Environmental Studies, Onogawa, Tsukuba, Ibaraki, Japan

3) Lake Biwa Environmental Research Institute, Otsu, Shiga, Japan

4) Graduate School of Science & Engineering, Soka University, Tokyo, Japan

*author for correspondence: kazama.takehiro@nies.go.jp (TK)

Running head: Carbon fixation model using multi-excitation wavelength FRRf in Lake Biwa

Subject area: Environmental science, Field ecology, Fluorometry, Limnology

Tables: 5

Figures: 6

Supporting information: S1-S4 Table, S1-S5 Appendix

No. of words in the main text: 6288

No. of words in Abstract: (max 300): 285

No. of references: 107

E-mails: TK, kazama.takehiro@nies.go.jp; KH, hayakawa-k@lberi.jp; KS, shimotori.koichi@nies.go.jp; VSK, victor@soka.ac.jp; AI, aimai@nies.go.jp; KK, kkomatsu@nies.go.jp

Abstract

Direct measurements of gross primary productivity (GPP) in the water column are essential, but can be spatially and temporally restrictive. Fast repetition rate fluorometry (FRRf) is a bio-optical technique based on chlorophyll *a* (Chl-*a*) fluorescence that can estimate the electron transport rate (ETR_{PSII}) at photosystem II (PSII) of phytoplankton in real time. However, derivation of phytoplankton GPP in carbon units from ETR_{PSII} remains challenging because the electron requirement for carbon fixation ($\Phi_{e,C}$) can vary depending on multiple factors. Also, the FRRf is still relatively novel, especially in freshwater ecosystems where phosphorus limitation and cyanobacterial blooms are common. The goal of the present study is to construct a robust $\Phi_{e,C}$ model for freshwater ecosystems using simultaneous measurements of ETR_{PSII} by FRRf with multi-excitation wavelengths coupled with traditional carbon fixation rate by the ^{13}C method. The study was conducted in oligotrophic and mesotrophic areas in Lake Biwa from July 2018 to May 2019. The combination of excitation light at 444, 512 and 633 nm correctly estimated ETR_{PSII} of cyanobacteria. The range of $\Phi_{e,C}$ in the phytoplankton community varied from 1.1 to 31.0 mol e^{-} mol C^{-1} during the study period. Generalized liner model showed the best model including 12 physicochemical and biological factors explained 67% of the variance in $\Phi_{e,C}$. Among all factors, water temperature was the most significant, while PAR intensity was not. The GPP values estimated by FRRf

(GPP_f) with the best $\Phi_{e,C}$ model relative to ^{13}C (GPP_{13C}) varied 0.5–1.5. Further, GPP_f estimated with more parsimonious $\Phi_{e,C}$ models were also comparable to GPP_{13C}. This study quantifies the applicability of the *in situ* FRRf methodology, and supports continuous monitoring of GPP by FRRf in lakes with large spatio-temporal variability of environmental conditions and phytoplankton assemblages. (285 words)

Introduction

Phytoplankton are the most important primary producers in the aquatic food web [1]. Changes in phytoplankton primary productivity can affect the food chain length [2,3], material cycles [4,5], and biomass of higher trophic organisms [6–8]. Phytoplankton community productivity is affected and must rapidly adapt to various environmental factors [9–11] due to rapid growth rates and short generation times [12]. In order to evaluate the effect of variability in environmental factors on aquatic communities and ecosystems, continuous observation of phytoplankton primary productivity is necessary [4,7,13].

Traditional chemical methods of primary production measurements, *i.e.*, the ^{14}C method [14], the ^{13}C method [15,16], the light-dark bottle method [17], and the ^{18}O method [18], require handling radioisotope (^{14}C) and/or incubation time for several hours. Thus, primary production studies using these techniques can be limiting when trying to assess temporal and spatial variability. Fast repetition rate fluorometry (FRRf; Table 1), a chlorophyll *a* fluorescence-based method, has been developed as an advanced bio-optical technique for real-time measurement of phytoplankton primary productivity, mainly in marine ecosystems [19–27]. The FRRf method enables induction and measurement of a range of chlorophyll *a* fluorescence yields and parameters specific to photosystem II (PSII) [19,20,28],

and, in turn, enables estimation of the *in vivo* electron transport rate in PSII (ETR_{PSII}) and gross primary productivity (GPP) by theoretical models of photosynthesis [19,28,29].

Table 1. Terms used within this manuscript.

Term	Definition	Units
a ($_{CDOM}$, $_{NAP}$, $^{*}_{phy}$ or $_{w}$)	Absorption spectrum (of CDOM, NAP, phytoplankton or pure water)	m^{-1} or m^{-2} mg Chl-a^{-1}
AEF	Alternative electron flow	
E ($_{FRRf}$, $_{0}$, $_{z}$ or $_{in\ situ}$)	Incident photosynthetically active radiation between 400 and 700 nm (of excitation flashlets, or at 0, z m or *in situ*)	μmol photons m^{-2} s^{-1}
$ETR_{(PSII)}$	Electron transport rate (in PSII)	
FRRf	Fast Repetition Rate Fluorometry	
GLM	Generalized linear model	
GPP	Gross primary productivity per area	g C m^{-2} d^{-1}

$\Phi_{e,C}$	Electron requirement for carbon fixation, also called *Kc*	mol e^{-} mol C^{-1}
K_d	Extinction coefficient of light	m^{-1}
PSII	Photosystem II	
RCII	PSII reaction centers	nmol m^{-3}
SCF	Spectral correction factor	
**Fluorometry**		
C	Fraction of RCII in closed state	
F'	Fluorescence at zeroth flashlet of a single turnover measurement when C>0	
F_O (′)	Minimum PSII Fluorescence yield (under background light)	
F_v (′)	$F_m(')-F_O(')$	
F_m (′)	Maximum PSII Fluorescence yield (under background light)	

F_v/F_m	Maximum photochemical efficiency under dark conditions	
GPP_f	GPP estimated by FRRf	g C m^{-2} d^{-1}
J_f	RCII-specific electron transport rate in PSII based on FRRf	μmol e^{-} nmol $RCII^{-1}$ s^{-1}
JV_f	Electron transport rate per water volume in PSII based on FRRf	μmol e^{-} m^{-3} s^{-1}
NPQ_{NSV}	Non-photochemical quenching based on normalized Stern-Volmer quenching coefficient	
qP	Separate package model of connectivity between RCIIs	
$R\sigma_{PSII}$ (′)	Probability of an RCII being closed during the first flashlet of a single turnover saturation phase (under background light)	
σ_{PSII} (′)	Functional absorption cross section of PSII for excitation flashlets (under background light)	nm^2

**^{13}C method**		
GPP_{13C}	GPP estimated by ^{13}C	g C m^{-2} d^{-1}
GP_C	Gross primary productivity per water volume based on ^{13}C	mg C m^{-3} h^{-1}
PB_C	RCII-specific primary productivity based on ^{13}C	mg C nmol $RCII^{-1}$ h^{-1}
**Light-dark bottle method**		
JV_O	Electron transport rate per water volume based on light-dark bottle	μmol e^{-} m^{-3} s^{-1}
NPo	Net oxygen evolution rate based on light-dark bottle	mg O_2 m^{-3} h^{-1}
R_d	Dark respiration rate based on light-dark bottle	mg O_2 m^{-3} h^{-1}

Previous studies demonstrated that GPP estimated from FRRf measurements correlated well with results from conventional methods, including the ^{13}C method [25,26,30,31] and the light-dark bottle method [24]. However, FRRf tended to over- and under-estimate GPP compared to the ^{14}C and ^{13}C methods, and the light-dark bottle and ^{18}O methods, respectively [32]. These discrepancies in GPP

measurements are dependent on the targeted products, *i.e.* oxygen or particulate organic carbon, in the photosynthesis cycle [33]. In order to account for the measurement discrepancies, recent studies are examining the electron requirement for carbon fixation ($\Phi_{e,C}$, also called *Kc*) by comparing the FRRf-derived ETR_{PSII} to the GPP rate by traditional methods [25–27,34]. The $\Phi_{e,C}$ is affected by multiple spatio-temporal variations in physical and chemical factors [35–40], and by phytoplankton community composition [26,41–44]. For example, $\Phi_{e,C}$ is relatively higher in the open ocean compared to coastal areas due to differences in the light environment conditions; light availability is higher in open oceans [35]. More specifically, excess light energy enhances photo-oxidative damage and alternative electron transport such as the Mehler reaction that can increase $\Phi_{e,C}$ [45]. In addition to ambient light conditions, nutrients can also play an important role in determining $\Phi_{e,C}$ of the phytoplankton community [27,39]. For example, Schuback et al. [27] described the negative relationships between $\Phi_{e,C}$ and nitrate concentrations in the Arctic Ocean suggesting the variable effects of nutrient availability. The multitude of interacting factors affecting the value of $\Phi_{e,C}$ for converting the ETR_{PSII} to GPP make it difficult to establish a general model applicable to different ecosystems [35]. Therefore, in order to construct a robust ETR_{PSII}-GPP model, it is necessary to accumulate FRRf and its corresponding GPP data in various aquatic environments.

In terms of physicochemical and biological conditions, freshwater ecosystems differ considerably from marine ecosystems. For example, cyanobacteria (blue-green algae) can frequently form dense surface blooms in meso−eutrophic lakes [46,47]. Previous studies have suggested cyanobacterial blooms can significantly affect ETR_{PSII} measurements due to spectral mismatch between FRRf excitation wavelengths and the absorption spectrum of cyanobacteria [21,26,36,41,48]. For example, Raateoja et al. [41] found that filamentous cyanobacteria *Nodularia spumigena* and *Aphanizomenon* sp. had absorption peaks around 630 nm, and the photosynthetic activity of these species could not be measured by FRRf with an excitation light around 475 nm (targeting Chl-*a*). Cyanobacteria have multiple absorption peaks around 500–570 nm and 630 nm based on antenna pigments [49]. Thus, it is critical that the FRRf excitation wavelengths correspond to the absorption spectrum of cyanobacteria (or the dominant group) for accurate estimation of primary productivity and model development in freshwater ecosystems [21,41,50].

Phytoplankton primary productivity is also more likely to be phosphorus-limited in freshwater ecosystems [51,52], while more likely to be nitrogen-limited in marine environments [53,54]. Whereas nitrogen limitation depresses cellular Chl-*a* concentration, phosphorus limitation inhibits ATP synthesis, which can affect photochemical energy conversion in algae [55]. In fact, $\Phi_{e,C}$ of marine

phytoplankton increases under nitrogen limitation [27,39]. Regrettably, phosphorus stoichiometry influence of $\Phi_{e,C}$ in freshwater phytoplankton remains unknown, and FRRf studies in freshwater environments in general are still limited [56–58]. Due to the differences between marine and freshwater ecosystems, it is essential to employ suitable excitation wavelength combinations and measure phosphorus concentration for correctly estimating both the ETR_{PSII} and GPP of freshwater phytoplankton communities.

The goal of the present study is to construct a robust $\Phi_{e,C}$ model applicable to freshwater ecosystems using simultaneous measurements of ETR_{PSII} by FRRf coupled with traditional carbon fixation rate by the ^{13}C method in Lake Biwa. From this context, we first evaluated the performance of FRRf with multi-excitation wavelengths (444, 512, and 633 nm; S1 Appendix A) during cyanobacterial blooms. Then, we evaluated the relative importance of variable environmental and biological factors, including phosphorus concentration, by determining $\Phi_{e,C}$ by statistical models with multiple variables. This study shows the utility of *in situ* FRRf measurements using excitation wavelength 633 nm during cyanobacterial blooms, and the extent to which physicochemical factors and phytoplankton community composition influence $\Phi_{e,C}$ estimation.

Materials and methods

Study site

The study was conducted at Lake Biwa (670 m^2 surface area with a mean depth of 43 m) on Honshu Island, Japan (Fig. 1). The North Basin is a deep, oligotrophic area, while the South Basin is a shallow, mesotrophic area [59]. Phytoplankton communities are substantially different between the two basins, especially in terms of cyanobacteria abundance [60]. Sampling was carried out at the long-term survey stations, 12B (62 m depth) and 17B (89 m depth) in the North Basin, and at 6B (4 m depth) and 9B (5.6 m depth) in the South Basin, from July 2018 to May 2019 (Table 2).

Table 2. Sampling date, Station ID, light environment (E_0 and K_d), and time periods for FRRf and ^{13}C incubation on each sampling date. E_0: sub-surface PAR at 0 m at the time of FRRf sampling; K_d: diffuse attenuation coefficient of PAR calculated from equation (1). Additional calculations of K_d were made for each layer when the logarithmic slope significantly changed with depth.

Date	Site	E_0 μ mol m^{-2} s^{-1}	K_d m^{-1}	FRRf periods	^{13}C incubation periods	Light-dark bottle incubation periods
Jul 23, 2018	12B	1630	0.433	10:30–10:50	14:00-17:00	

Jul 30, 2018	17B	705	0.328	11:10–11:30	14:30–17:30	
Aug 28, 2018	9B	650	1.727 (0–2 m)	11:00–11:20	15:00–18:00	15:00–18:00
			0.971 (2–5 m)			
Sep 13, 2018	12B	440	0.541 (0–6 m)	10:15–10:35	14:30–17:30	14:30–17:30
			0.261 (6–20 m)			
Sep 18, 2018	6B	124	1.029	9:35–10:00	14:15–17:15	14:15–17:15
Oct 10, 2018	9B	176	1.001	10:40–10:50	14:45–17:45	
Oct 25, 2018	9B	1090	0.881	10:00–10:15	14:30–17:30	
Nov 16, 2018	12B	901	1.198 (0–2 m)	9:45–10:00	14:00–17:00	
			0.375 (2–20 m)			
Dec 10, 2018	6B	221	1.117	10:30–10:45	14:45–17:45	
Dec 19, 2018	17B	255	0.451 (0–6 m)	11:00–11:30	16:00–19:00	
			0.224 (6–20 m)			
Jan 28, 2019	9B	235	1.029 (0–2 m)	10:30–10:45	14:30–17:30	
			0.387 (2–4.5 m)			
Feb 7, 2019	12B	279	0.536 (0–2 m)	9:35–10:00	14:30–17:30	

			0.252 (2–20 m)		
Apr 19, 019	9B	445	1.916 (0–1 m)	10:40–11:00	14:00–17:00
			0.607 (1–5.5 m)		
May 10, 2019	12B	1711	0.894 (0–2 m)	10:10–10:30	15:00–18:00
			0.263 (2–20 m)		

Fig. 1. Map of study sites in Lake Biwa, Japan. Stations 6B and 9B represent the South Basin, while Stations 12B and 17B were selected as representatives of the North Basin. Grey dotted lines indicate the isobaths (in m), and dashed line represents the boundary of the basin.

Sampling procedure

Vertical profiles of water temperature, pH, dissolved oxygen concentration, and specific conductivity were measured using a water quality sonde (EXO2; Xylem, Inc., Yellow Springs, OH, USA). At stations 12B and 17B, water samples were collected with a bucket at 0 m, with 10-L Niskin samplers at 2.5 m, and with 5-L Niskin bottles on a rosette sampler (AWS, JFE Advantech Co. Ltd., Kobe, Japan) at 5, 10, 15, and 20 m after *in situ* FRRf measurement (described below). Likewise, at

stations 6B and 9B, water samples were collected with a bucket at 0 m, and with an electric pump at 2 and 3 m (6B) or 4 m (9B).

Macro-nutrients and dissolved inorganic carbon (DIC) concentrations were determined from an aliquot of 100 mL subsample collected at each depth, and immediately filtered through a syringe-type membrane filter (0.2 µm pore size, Acrodisc syringe filter; Pall Corporation, Ann Arbor, MI, USA) using clean techniques. The filtered samples were stored at −20°C until nitrate, nitrite, and phosphate analyses using an ion chromatograph system (Dionex Integrion HPIC system; Thermo Scientific, Waltham, MA, USA). DIC was analyzed using a total carbon analyzer (TOC-L; Shimadzu, Kyoto, Japan). For chlorophyll *a* (Chl-*a*) analysis, 50–200 mL sample was filtered onto a 25-mm glass-fiber filter (0.7 µm nominal pore size, GF/F; GE Healthcare, U.K. Inc., Little Chalfont, England). Chl-*a* was extracted with *N*,*N*-dimethylformamide for 24 h in the dark [61] and then stored at −80°C. The Chl-*a* concentration was determined with a 10-AU fluorometer (Turner Designs, Sunnyvale, CA, USA).

We measured underwater photosynthetically active radiation (PAR, 400–700 nm) from 30 m to the surface using a 2 π PAR sensor (CTG Ltd, West 106 Molesey, UK) along with the FRR fluorometer. We determined the diffuse attenuation coefficient K_d (m^{-1}) with an exponential function as follows:

$$E_Z = E_0 \exp(-K_d \times Z) \quad (1)$$

where E_Z and E_0 are incident PAR (μmol photon m^{-2} s^{-1}) at depth, Z, and 0 m, respectively. When the logarithmic slope of K_d significantly changed with depth, we calculated it for each layer (Table 2).

FRRf measurements and photophysiological parameters

In situ induced Chl-*a* fluorescence profiles were measured vertically with a multi-excitation wavelength Fast Repetition Rate fluorometer (FRRf) system (FastOcean, S/N 17-0053-002; CTG Ltd, West 106 Molesey, UK). The field-type FRRf was equipped with two chambers for ambient light and dark readings. In order to remove ambient light noise, an optical bandpass filter (<670 nm) was attached above the light chamber. The dark chamber has black housing and piping with a pump to ensure that samples are measured under complete dark after 1–2 s of dark adaptation. Each chamber has three light-emitting diodes (LED) providing flash excitation energy centered at 444, 512, and 633 nm (S1 Appendix A). The 444 nm (blue) corresponds to the absorption peak of Chl-*a* while 512 nm (green) and 633 nm (orange) correspond to the absorption peaks of phycoerythrins and phycocyanins [49]. We employed four LED combinations to evaluate the green and orange excitation flashes: (1) 444 nm (2) 444 and 512 nm, (3) 444 and 633 nm, and (4) 444, 512 and 633 nm. We applied a single turnover method, which was consistent with a saturation phase (100 flashlets with 2 μs pitch) and a

relaxation phase (40 flashlets with 50 μs pitch). This sequence was repeated eight times with a 100-ms interval for each LED combination. All combinations were repeated at least five times every 5 m from 20 to 10 m and every 2 m from 10 m to the surface at 12B and 17B, and every 0.5 m from the bottom to the surface layer at 6B and 9B during the up-cast, respectively. The power of flashlets (E_{FRRf}) and the gain of the extra high tension of the photomultiplier tube (PMT eht) were optimized by FastPro8 software (version 1.0.50; CTG Ltd). All FRRf measurements were done between 09:30 and 11:30 (Table 2).

The concentration of PSII reaction center (RCII, nmol m^{-3}) was estimated fluorometrically according to Oxborough et al. [28] as follows:

$$\text{RCII} = K_R/E_{FRRf} \times F_o/\sigma_{PSII} \times 10^{-9} \quad (2)$$

where K_R is an instrument-specific constant (photons m^{-3} s^{-1}), F_o is the fluorescence intensity at the zeroth flashlet of a single turnover measurement when all RCII are open, and σ_{PSII} is the absorption cross section of PSII photochemistry under dark (m^2). A recent study showed K_R/E_{FRRf} can vary among phytoplankton taxonomic group and growth condition, and affect estimation of productivity [62]. In this study, we did not examine sample-specific K_R/E_{FRRf} values. Instead, we used a constant value as in Wei et al. [63], and taxonomic group and nutrients were assessed as the factors affecting $\Phi_{e,C}$ by

statistical model approach (mentioned below).

The RCII-specific rate of electron transport based on FRRf (J_f, μmol electrons nmol RCII^{-1} s^{-1}) was calculated based on the Sigma Algorithm installed in FastPro8 [28,36]:

$$J_f = E \times \sigma_{PSII}' \times (1 - C) \quad (3)$$

where E is the incident PAR at each sampling depth (μmol photon m^{-2} s^{-1}), σ_{PSII}' is the absorption cross section of PSII photochemistry under ambient light (m^2), and $(1 - C)$ is the fraction of RCII in the open state which is assumed, qP (=($F'-F_O'$)/($F_m'-F_O'$)). Thus, the electron transport rate per water volume (JV_f, μmol electrons m^{-3} s^{-1}) was derived by $J_f \times$ RCII

Phytoplankton can dissipate excessive energy as heat in PSII, and after PSII through alternative electron flows (AEFs) such as the Mehler reaction (for reviews, see [33,64]). Among these, the proportion of heat dissipation in total absorbed energy was estimated as the normalized Stern–Volmer quenching coefficient (NPQ_{NSV}), which is equal to F_O'/F_v' [65]. This is commonly used to compare non-photochemical quenching among phytoplankton communities that have different light history [27,38,66]. Finally, the maximum quantum efficiency of PSII was evaluated by F_v/F_m [67].

Evaluation of excitation wavelength combination

In order to assess the performance of the four wavelength combinations in the natural phytoplankton communities, we compared the minimum fluorescence yield *Fo* measured at each wavelength combination during cyanobacterial blooms (at 9B on August 28 in 2018), and during diatoms and zygnematophytes dominated (at 12 B on September 13 and at 6B on September 18 in 2018). We also compared electron transport rates estimated by FRRf (JV_f, μmol e^- m^{-3} s^{-1}) and by light-dark bottle method (JV_O, μmol e^- m^{-3} s^{-1}) [68] to verify the accuracy of estimated JV_f.

The light-dark bottle method has been known to underestimate GPP due to differences in respiration rate between dark and light conditions [69,70], and oxygen oversaturation [71]. Nevertheless, since one molecule of CO_2 fixation theoretically requires at least four electrons through photosynthesis, the method is still practical to determine whether the JV_f determined by the FRRf was underestimated, or not. Water samples from each layer were poured into two or four 100-mL glass bottles. To measure the community respiration rate, another aliquot of sample water from each layer was poured into two 100-mL dark bottles. All bottles were incubated for 3 h in a growth-chamber (HCLP-880PF, Nippon Medical and Chemical Instruments Co., Ltd., Japan). Oxygen concentration of each bottle was measured by the optical oxygen spots and probe (Fibox 4; PreSens, Regensburg, Germany) before and after incubation. JV_O was derived as

$$JV_O = (NP_O - R_d) \times 3.47 \times 10^{-2} \quad (4)$$

where NP_O is net oxygen evolution rate (mg O_2 m^{-3} h^{-1}), R_d is dark respiration rate (mg O_2 m^{-3} h^{-1}), and 3.47×10^{-2} is a conversion factor from hour to seconds, from mg O_2 to µmol O_2, and 4 mol e^- for 1 mol O_2 evolution. Plots of JV_O against light intensity were fitted in a two-parameter model as described in Webb et al. [72].

Additionally, quality control of all FRRf data measured with each excitation combination was assessed by the probability of an RCII being closed during the first flashlet of a single turnover saturation phase under dark ($R\sigma_{PSII}$) and ambient light ($R\sigma_{PSII}'$) by FastPro8 software. Although FastPro8 adjusted $R\sigma_{PSII}$ and $R\sigma_{PSII}'$ around 0.05, these values changed dependent on depth, light environment and phytoplankton community composition. Thus, we compared the number of successful observations, $R\sigma_{PSII}$ and $R\sigma_{PSII}'$ among 4 combinations after rejection of extremely low-quality data ($R\sigma_{PSII}$ or $R\sigma_{PSII}'$ <0.03 or >0.08).

Phytoplankton identification and enumeration

For enumerating phytoplankton, 50 mL of each sample was fixed with Lugol's solution (1% of final concentration). After 24 h of settling in the dark, the supernatant was removed gently, and the

sample was concentrated to 15 mL. Cells were counted under a light microscope at ×200 magnification where the size and volume of cells for each group were measured using cellSens software (Olympus, Tokyo, Japan) based on Hillebrand et al. [73]. All phytoplankton species were categorized into eight groups: diatoms, cyanophytes, small chlorophytes, zygnematophytes, cryptophytes, crysophytes, dinoflagellates, and euglenophytes. The phytoplankton community composition was assessed based on the carbon biomass converted from the biovolume [74].

^{13}C uptake rate

Gross primary production per water mass (GP_C, mg C m^{-3} h^{-1}) was determined by a ^{13}C-based method based on Hama et al. [16]. Water samples from each layer were taken to the laboratory and poured into two or four 500-mL polycarbonate bottles (Nalgene, Rochester, NY, USA), and spiked with $NaH_{13}CO_3$ to a final concentration of ca. 10% of ambient total inorganic carbon [75]. Incubations were initiated within 3 h after samples collected. Production experiments were conducted in incubators where the temperature and light environment were controlled in a growth-chamber along with an oxygen evolution experiment. Incubation PAR levels were set as follows: For site 12B and 17B, 100% and 65% of E_0 for 0 m samples, and 30%, 10%, and 1.6% of E_0 for 2.5-, 5- and 10-m samples; For site

6B, 100% and 65% of E_0 for phytoplankton for the 0-m sample, 30% and 10% of E_0 for the 2-m sample, and 1.6% of E_0 for the 3-m sample; For site 9B, it was same as site 6B but 1.6% of E_0 for the 4-m sample. Incubation PAR intensity was manipulated using black mesh filters covering polycarbonate bottles. Incubation temperature was set to the mean of respective sampling depths. The samples from 12B on 23 July and at 17B on 30 July were incubated under ambient light percentages (described above) and temperature on the balcony of Lake Biwa Environmental Research Institute (Shiga, Japan). Although incubation temperature was not controlled on 23 and 30 July, it changed <1.5°C during the incubations.

All incubations were conducted for 3 h. After each incubation period, water samples were filtered through pre-combusted (at 450°C for 4 h) 25-mm glass-fiber filters (0.7 μm nominal pore size, GF/F). The filters were stored at −20°C until final analysis. The carbon stable isotope ratio $\delta^{13}C$ was measured using Delta V Advantage isotope ratio mass spectrometer coupled with Conflo IV interface and Flash 2000 elemental analyzer (Thermo Fisher Scientific, Waltham, MA USA) at the Isotope Research Institute (Yokohama, Japan). GP_C was calculated according to Hama et all [16]. To compare with the measured J_f in equation (3), GP_C was converted to RCII-specific primary production rate (PB_C, mg C nmol RCII^{-1} h^{-1}) as follows:

$$PB_C = \frac{GP_C}{RCII} \quad (5)$$

Spectral correction

In order to account for the difference in spectral distribution and primary production response between ambient light field and artificial incubator light sources, we applied a spectral correction to the data-set to reduce any possible discrepancies between the methods. First, to correct the differences in the spectral distribution of excitation flash of FRRf and ambient light in water column, σ_{PSII} was adjusted by spectral correction factor (SCF) following previous studies [27,34]:

$$SCF = \frac{\sum_{700}^{400} a_{phy}^{*}(\lambda)\times E_{in\,situ}(\lambda)\sum_{700}^{400} E_{FRRf}(\lambda)}{\sum_{700}^{400} a_{phy}^{*}(\lambda)\times E_{FRRf}(\lambda)\sum_{700}^{400} E_{in\,situ}(\lambda)} \quad (6)$$

where $a_{phy}^{*}(\lambda)$ is the Chl-*a* specific absorption spectrum of phytoplankton (m^2 mg Chl-a^{-1}), $E_{in\,situ}$ (λ) and E_{FRRf} (λ) are the spectral distribution of irradiance in the water column and excitation flash of FRRf, respectively. The $a_{phy}^{*}(\lambda)$, and $E_{in\,situ}$ (λ) were estimated from models described in previous studies where the spectral irradiance in the water column was estimated as follows [76,77],

$$E_{in\,situ}(\lambda, z) = E_0(\lambda)exp(-\left[a_W(\lambda) + a_{CDOM}(\lambda) + a_{NAP}(\lambda) + chl \times a_{phy}^{*}(\lambda)\right] \times z) \quad (7)$$

where λ is wavelength between 400 to 700 nm, a_W, a_{CDOM}, and a_{NAP} are absorption spectrum of pure water, CDOM, and non-algal particles, respectively (m^{-1}), and *chl* is Chl-*a* concentration (mg m^{-3}) and

z is depth (m). $a^*_{phy}(\lambda)$ was estimated for each date and depth by Paavel's model [78] for August, and Ylöstalo's model [79] for other months in accordance with the species composition of phytoplankton community (S2 Appendix).

The a_W was estimated by a common spectra model determined by Pope and Fry [80] (S3 Appendix A). The a_{CDOM} was estimated by the equation a_{CDOM} = a_{CDOM} (320) exp(-S(λ-320)) [81]. We used previously measured, average values of limnetic sites from each basin, 1.03 and 2.28, for a_{CDOM} (320) for the North Basin and the South Basin, and 0.017 for S [77,81] (S4 Appendix B). a_{NAP} was estimated as a_{NAP} = a_{NAP} (440) EXP(-S (λ-440)) [82]. We used 0.264 for a_{NAP} (440) and 0.004 for S as typical values for the area (S4 Appendix C). The calculated $E_{in\ situ}(\lambda, z)$ was adjusted with observed PAR intensity at each depth during each sampling date.

For spectral distribution of incident sunlight $E_0(\lambda)$ on each sampling date, we referred to the solar radiation spectrum database of central Japan in 2015 [83]. In order to fit the incident angle of sunlight and the weather condition on each sampling date, we used spectral data at 10 AM on January 30, February 9, April 8, May 17, July 2, 31, August 26, September 7, 19, October 10, 26, November 10, December 9 and 19 corresponding to each sampling date (S4 Appendix). For spectral irradiance during ^{13}C incubation on July 23 and 30, we used spectral data at 4 PM on July 2 and 31 from the database.

Calculated SCFs are listed in the S1 Table in supporting information. The SCF for PAR intensity of each growth-chamber was also adjusted with the spectral distribution of the light source (S1 Appendix B) and $a^*_{phy}(\lambda)$ in the same manner as equation (6).

Derivation of photosynthetic parameters

In order to calculate $\Phi_{e,C}$ (mol e^- mol C^{-1}) from J_f and PB_C assessed at different light levels, photosynthesis versus irradiance curves (*P-E* curves) were obtained by curve-fitting using two *P-E* models. When J_f or PB_C showed photoinhibition (*i.e.*, decreasing J_f or PB_C with increasing E after the light-saturated phase), *P-E* curves were fitted in a three-parameter model as described in earlier studies [84–86]. When there was no photoinhibition, *P-E* curves were fitted in a two-parameter model as described in Webb et al. [72].

It should be noted that the phytoplankton community structures were occasionally different among the layers of water column, but the relationships between J_f or PB_C and E were well-fitted in the photosynthesis models, as in previous studies [25,30]. All parameters in the fitted models were calculated by function nls() in R ver. 3.4.3 [87]. The PB_C values corresponding to underwater E of *in situ* FRRf measurements were extracted from the *P-E* curves. PB_C can be functioned by J_f with electron requirement for carbon fixation ($\Phi_{e,C}$) as

$$PB_c = J_f / K_c \times 43.2 \quad (8)$$

where 43.2 is the conversion factor from seconds to hour, and from μmol C to mg C, respectively.

Data transformation and GLM for $\Phi_{e,C}$

Because FRRf measurements were inhibited by high light intensity (typically >1,000 μmol photons m^{-2} sec^{-1}), presumably there were sampling biases between shallow and deep layers. To avoid this bias, we subsampled 240 observations from the data set on each sampling date by bootstrap sampling with replacements; 60 observations from four layers above the euphotic zone (0–3.75, 3.75–7.5, 7.5–12.5, and 12.5–17.5 m) for the North Basin and 80 observations from three layers (0–1, 1–3 and 3–5.5 m) for the South Basin. Here a total of 3360 observations (1680 observations for the North Basin and 1680 observations for the South Basin) were used in all analyses of this study.

As in equation (8), $\Phi_{e,C}$ is defined as $J_f / PBc \times 43.2$. We modeled $\Phi_{e,C}$ using a generalized linear model (GLM) with gamma error distribution and a log-link function using the glm() function in R. We treated $\Phi_{e,C}$ as the dependent variable, and environmental factors as the explanatory variables. To avoid collinearity between the explanatory variables, Spearman's ρ between all candidate factors were tested with a significance level of $p < 0.05$, and parameters with $\rho \geq 0.7$ were regarded as collinear variables.

We selected water temperature, PAR, turbidity, dissolved oxygen (DO), NH_4, NO_3 + NO_2, PO_4, F_v/F_m, σ_{PSII}, Chl-*a*, and the fractions of diatom, cyanobacteria, and cryptophytes in phytoplankton community as the explanatory variables in GLM. Explanatory variables were standardized (mean 0 and standard deviation 1) after log-transformation. NH_4 concentration and the fraction of phytoplankton group biomass lower than the detection limit were treated as 0.1 μmol L^{-1} and 0.1%, respectively. Multicollinearity of variables were further tested with the variance inflation factor (VIF) [88] using vif() in the package 'car' [89] in R. A set of all possible submodels was generated with the dredge() function in package 'MuMIn' [90], and the submodels were ranked based on the Akaike information criterion (AIC) [91].

Results

Evaluation of excitation wavelength combination

Vertical profiles of temperature showed weak stratification at Station 9B on August 28 and at Station 12B on September 13 (Fig. 2A, D) but not at Station 6B on September 18 (Fig. 2G). Chl-*a* concentration reached 42 μg L^{-1} at 2 m at Station 9B due to cyanobacterial bloom (Fig. 2C). Vertical profiles of the minimum PSII fluorescence yield (*Fo*) showed variability between four combinations of

excitation wavelengths during cyanobacterial blooms (Fig. 2B), but not for diatom and zygnematophytes dominant communities (Fig. 2E, H). For example, *Fo* profiles derived by excitation lights at 444 nm and 444 + 512 nm were lower than excitation combinations at 444 + 633 nm and 444 + 512 + 633 nm when cyanobacteria were dominant at 0 and 2 m depth at Station 9B (South Basin) in August (Fig. 2B, C). On the other hand, there were no clear differences in *Fo* profiles at Station 12B (North Basin) on September 13 and Station 6B (South Basin) on September 18 when diatoms and zygnematophytes dominated (Fig. 2E, F, H, I). Further, the relationship between JV_f and PAR intensity shows the utility of 633 nm for revealing signatures of cyanobacteria photosynthesis (Fig. 3). For example, JV_f was clearly lower than JV_O using excitation at 444 nm and 444 + 512 nm, but not at 444 + 633 nm and the combination of all three wavelengths at Station 9B on August 28 during a cyanobacteria bloom (Fig. 3A). No clear differences in JV_f were observed between the combinations of wavelengths at Station 12B on September 13 and Station 6B on September 18, 2018 (Fig. 3B, C).

The data quality of FRRf measurements during the study period showed reasonable stability (Table 3). Upon rejection of low-quality data (*e.g.*, with $R\sigma_{PSII}$ or $R\sigma_{PSII}'$ < 0.03 or > 0.08), the number of successful observations was found to be highest when PSII was excited with a combination of three wavelengths, followed by excitation light at 444 + 633 nm, 444 + 512 nm, and 444 nm. Median values

of both $R\sigma_{PSII}$ and $R\sigma_{PSII}'$ were also near the optimal value (0.05) when the three wavelengths were combined.

Table 3 Comparison of the probability of RCII being closed during the first flashlet of a single turnover saturation phase under dark ($R\sigma_{PSII}$) and ambient light ($R\sigma_{PSII}'$) among four combinations of excitation wavelength. N, number of successful observations.

Excitation wavelength	N	$R\sigma_{PSII}$		$R\sigma_{PSII}'$	
		Median	Range	Median	Range
444 nm	1419	0.036	0.030–0.050	0.037	0.030–0.075
444 +512 nm	1868	0.039	0.030–0.055	0.041	0.030–0.071
444 +633 nm	1875	0.041	0.030–0.055	0.043	0.030–0.070
444 +512 +633 nm	1925	0.045	0.030–0.061	0.048	0.030–0.078

Fig. 2. Vertical profiles of PAR, water temperature and Chl-*a* (A, D, G), *Fo* (B, E, H) estimated by different combinations of excitation wavelength from the FRRf, and phytoplankton biomass (C, F, I) at Station 9B in August 28 (A, B, C), Station 12B in September 13 (D, E, F) and

Station 6B in September 18, 2018 (G, H, I). Grey dashed lines denote 0 m.

Fig. 3. Scatter plots of JV_f estimated by different combinations of excitation wavelength from the FRRf relative to ambient PAR intensity at (A) Station 9B on August 28, (B) Station 12B on September 13 and (C) Station 6B on September 18. The JV_O estimates from the light-dark bottle method are also shown. For JV_O, PAR intensity was calculated by light intensity of growth chambers and SCF (see Materials and methods). The fitted curve is given for JV_O using a two-parameter model [72] to improve visibility.

Development of $\Phi_{e,C}$ model

Environmental and biological conditions

Ancillary measurements of water temperature, DO concentration, turbidity, Chl-*a*, NO_2 + NO_3, NH_4 and PO_4 concentrations from each sampling showed clear spatial and seasonal variability (Table 4). Water temperature varied from 7.5 to 30.2°C in the North Basin and 7.5 to 28.5°C in the South Basin throughout the study period (Table 4). NO_2 + NO_3 and NH_4 concentrations were lower in summer and autumn, and higher in winter at both basins throughout the study period. PO_4 did not show clear

seasonal changes and was always lower than 0.04 μmol L^{-1} in both basins throughout the study period.

At all sampling dates, diatoms, zygnematophytes, cyanobacteria, and cryptophytes were the dominant groups in the phytoplankton biomass (S5 Appendix). Zygnematophytes, mainly composed of *Staurastrum dorsidentiferum*, *S. sebaldi*, and *Micrasterias hardyi*, were always found at all sites throughout the study period, except at Station 6B in December. Diatoms were dominant during summer to early autumn and reached 87% in the phytoplankton biomass at Station 6B in September (S5 Appendix). Cryptophytes were present at a relatively low proportion through the study period except at Station 6B in December. Cyanobacteria were mainly composed of *Anabaena* (*Dolichospermum*) *affinis* and *Aphanothece* sp. and bloomed at Station 9B in August 28. Small chlorophytes, crysophytes, and dinoflagellates always made up less than 20% of the total phytoplankton biomass. Euglenophytes were very rare and accounted for less than 0.5% of the total biomass through the study period.

Table 4. Physical, chemical and biological (ancillary) conditions on sampling dates. The values denote depth ranges of 0 – 17.5 m for both Stations 17B and 12B, 0 – 4 m for Station 9B, and 0 – 3 m for Station 6B. NTU, nephelometric turbidity units; LOD, limit of detection.

Date	Site	Temperature	DO	Turbidity	Chl-a	NO_2+NO_3	NH_4	PO_4

		(°C)	(%)	(NTU)	(µg L^{-1})	(µmol L^{-1})	(µmol L^{-1})	(µmol L^{-1})
Jul 23, 2018	12B	17.8–30.2	82–116	0.3–1.3	2.4–13.0	0.1–1.1	0.2–0.4	0.01–0.03
Jul 30, 2018	17B	22.2–27.7	87–103	0.2–0.9	2.4–12.8	0.1–0.5	0.2–0.2	0.01–0.03
Aug 28, 2018	9B	25.0–28.5	79–136	1.6–8.2	8.0–41.9	0.4–1.1	0.8–1.1	0.02-0.02
Sep 13, 2018	12B	19.9–23.7	80–100	0.4–1.1	2.4–9.6	1.3–5.9	0.2–1.0	0.01–0.04
Sep 18, 2018	6B	24.2–24.4	102–103	2.2–4.3	7.6–12.4	0.3–1.7	1.0–1.2	0.01–0.02
Oct 10, 2018	9B	22.1–22.6	105–117	1.3–2.8	14.4–17.0	0.4–1.4	0.9–1.3	0.01–0.02
Oct 25, 2018	9B	19.6–19.8	107–110	1.0–1.9	16.2–17.3	0.1–0.4	0.8–0.8	0.01–0.03
Nov 16, 2018	12B	17.2–17.3	96–98	0.5–1.0	2.4–2.9	0.2–0.3	0.2–0.3	0.01–0.02
Dec 10, 2018	6B	11.5–11.6	98–99	2.2–2.7	7.6–8.0	0.1–0.5	0.8–0.9	0.03–0.03
Dec 19, 2018	17B	12.8–12.8	93–97	0.1–0.7	4.6–5.3	3.3–4.2	0.5–0.6	0.01–0.03
Jan 28, 2019	9B	7.5–8.3	96–98	1.1–2.6	2.5–3.4	4.8–5.9	1.0–1.2	0.02–0.03
Feb 7, 2019	12B	8.9–9.0	92–94	0.2–0.6	4.3–6.7	6.3–7.1	0.3–0.4	0.02–0.03
Apr 19, 019	9B	11.8–13.8	105–112	0.7–1.5	5.9–8.9	3.0–3.3	<LOD	0.01–0.03
May 10, 2019	12B	12.0–15.7	102–118	0.3–1.5	4.2–12.2	1.0–5.3	<LOD–0.8	0.01–0.03

Spatiotemporal variation and GLM development for $\Phi_{e,C}$

In order to develop an optimal electron requirement for carbon fixation ($\Phi_{e,C}$) model, we used the data set that was obtained by the combination of three excitation wavelengths due to the quality and reliability (Fig 2, 3). After bootstrap sampling, boxplots of median $\Phi_{e,C}$ were calculated for each sampling date (Fig. 4). $\Phi_{e,C}$ changed temporally from 1.1 to 31.0 mol e^{-} mol C^{-1} and were relatively higher in spring and summer in both the North and South basins. The mean annual $\Phi_{e,C}$ values were 5.6 mol e^{-} mol C^{-1} for the North Basin, 9.0 mol e^{-} mol $C^{-1}$for the South Basin, and 7.3 mol e^{-} mol C^{-1} for all sampling sites.

Fig. 4. Spatial and temporal variability of $\Phi_{e,C}$ of the phytoplankton community in the North Basin (A) and the South Basin (B) through the study period. The box plot shows the median (bold line), the 25th (Q1) and the 75th (Q3) percentile. The whiskers indicate 1.5 times the interquartile range (Q3−Q1) below and above the Q1 and Q3. Outliers beyond the whiskers were plotted individually. Note: $\Phi_{e,C}$ values were derived from the data measured by the combination of three excitation wavelengths.

In order to select and define the explanatory variables for GLM, we examined correlations between $\Phi_{e,C}$ and all candidate environmental factors (Fig. 5). Small chlorophytes, crysophytes, and dinoflagellates were excluded because of their low proportion in the total phytoplankton biomass. $\Phi_{e,C}$ correlated positively with PAR, temperature, DO, NPQ_{NSV}, Chl-*a*, and σ_{PSII}, and negatively with maximum photochemical efficiency under dark conditions (F_v/F_m) throughout the study period. NPQ_{NSV} highly correlated with PAR and F_v/F_m (ρ = 0.70 and −0.96, respectively). RCII concentration highly correlated with Chl-*a* (ρ = 0.70), and diatoms and zygnematophytes also negatively correlated (ρ = −0.70) with each other. Based on the correlation matrix, we selected temperature, PAR, turbidity, DO, F_v/F_m, σ_{PSII}, NH_4, NO_2 + NO_3, PO_4, Chl-*a*, and fractions of diatoms, cyanobacteria, and cryptophytes in the phytoplankton biomass as explanatory variables for the GLM. The effects of PAR and diatoms in this analysis may have included those of NPQ_{NSV} and zygnematphytes.

Among all possible models, the best model with the lowest AIC was the full model without PAR (Table 5). All variables in the best model exhibited VIF < 10, and thus, collinearity was negligible. The R^2 for the best model was 0.67. Among the explanatory variables, temperature showed the highest significance in the best model (coefficient of 0.51), followed by cyanobacteria (coefficient of −0.20), and σ_{PSII} (coefficient of 0.17). The performance of more parsimonious models were examined to

evaluate the laborious sampling effort of nutrients and microscopy analysis phytoplankton assemblages. The lowest AIC models without nutrients (Model 2), and without nutrients and phytoplankton assemblages (Model 3) were employed. The Model 2 included six variables, (temperature, F_v/F_m, σ_{PSII}, cyanobacteria, diatoms and cryptophytes), while Model 3 included three variables (temperature, F_v/F_m, and σ_{PSII}). The values for R^2 for the Model 2 and the Model 3 were 0.61 and 0.42, respectively. The results of the other sub-models with and without standardization of variables are listed in the supporting information (S2 Table and S3 Table).

Table 5. Statistical results of the GLM analysis showing the best model (smallest AIC in all models), the Model 2, and the Model 3 for $\Phi_{e,C}$. Coefficients were derived for log-transformed and standardized variables (see Materials and methods). AIC and R^2 for each model are also shown.

Variables	Coefficient	Std. Error	t value	*P*
**Best Model: AIC = 15481, R^2 = 0.67**				
Intercept	1.83	0.01	266.6	<0.001
Temperature	0.51	0.02	25.5	<0.001

DO	0.13	0.01	11.8	<0.001
Turbidity	0.10	0.01	8.0	<0.001
F_v/F_m	-0.10	0.01	-12.7	<0.001
σ_{PSII}	0.17	0.01	17.3	<0.001
PO_4	-0.02	0.01	-2.1	0.034
NH_4	-0.02	0.01	-2.7	0.006
NO_2+NO_3	0.13	0.01	12.0	<0.001
Chl-a	0.10	0.01	7.0	<0.001
Cyanobacteria	-0.20	0.01	-16.9	<0.001
Diatoms	-0.11	0.01	-10.2	<0.001
Cryptophytes	0.14	0.01	10.2	<0.001
**Model 2: AIC = 15987, R^2 = 0.61**				
Intercept	1.84	0.01	252.2	<0.001
Temperature	0.77	0.01	53.1	<0.001
F_v/F_m	-0.08	0.01	-10.7	<0.001
σ_{PSII}	0.10	0.01	12.1	<0.001

Cyanobacteria	-0.35	0.01	-32.9	<0.001
Diatoms	-0.04	0.01	-4.8	<0.001
Cryptophytes	0.28	0.01	25.2	<0.001
**Model 3: AIC = 17334, R^2 = 0.42**				
Intercept	1.89	0.01	193.2	<0.001
Temperature	0.28	0.01	25.5	<0.001
F_v/F_m	-0.15	0.01	-14.4	<0.001
σ_{PSII}	0.22	0.01	20.4	<0.001

Fig. 5. Matrix of Spearman's ρ between photosynthetic parameters measured by excitation wavelengths of 444 + 512 + 633 nm, physicochemical factors, and biomass fraction of each phytoplankton group. Colored panels denote statistical significance ($p < 0.05$). Abbreviations of variables are: Temp, water temperature; NTU, turbidity; Chl, Chl-*a* concentration; Diat, diatom; Cyano, cyanobacteria; Zyg, zygnematophytes; Cryp, cryptophytes.

Discussion

Recently, studies using FRRf with only one wavelength, around 450 nm, for primary production measurements have been successful [25,26]. However, the absorption spectrum of phytoplankton is highly dependent on the construction of antenna pigments, and is quite different between cyanobacteria and the other phytoplankton groups [92]. The present study demonstrated that JV_f measured with an excitation light of 444 nm (single source), or with excitation lights of 444 + 512 nm was considerably underestimated compared to measurements utilizing 633 nm, particularly when cyanobacteria dominated (Fig. 3). This underestimation is expected because of the mismatch in wavelength between excitation wavelengths of the FRRf and the absorption spectrum of cyanobacteria. The blue excitation flash at 444 nm can fail to saturate the RCII in cyanobacteria during a single turnover measurement of FRRf [41,93], and thus underestimate *Fo* [94] and GPP [21]. Although we estimated JV_f by each combination of excitation wavelength with SCF, which was calculated from extrapolated a^*_{phy}, there might be large differences between modeled and actual a^*_{phy} around 633 nm, but not around 512 nm in the green spectrum. Indeed, a^*_{phy} at 630 nm in August, when *Anabaena* spp. dominated, was estimated as 0.008−0.009 m^{-2} mg Chl-a^{-1} (S2 Appendix) while that of cultured *Anabaena* sp. was 0.028 m^{-2} mg Chl-a^{-1} [49]. Further, a^*_{phy} of cyanobacteria can vary with taxonomic group [49] and nutrient availability [95,96]. Our results suggest that if the absorption spectrum cannot be measured *in situ*, the

excitation light targeting phycobilin antenna pigments should be used to measure ETR_{PSII} in cyanobacterial dominant communities.

In this study, $\Phi_{e,C}$ in Lake Biwa ranged temporally from 1.1 to 31.0 mol e^{-} mol C^{-1} (Fig. 4). Although our $\Phi_{e,C}$ range varied less than those reported in temperate ocean conditions (1.0 to 66.5 mol e^{-} mol C^{-1} [26]), it was similar to that in an Atlantic Ocean transect (1.1 to 28.2 mol e^{-} mol C^{-1} [35]) and Ariake Bay (1.2 to 26.6 mol e^{-} mol C^{-1} [25]), and those in a shallow-eutrophic lake (from 14.7 to 38.6 mol e^{-} mol C^{-1} [58]). Considering that environmental conditions varied substantially among the sampling sites and seasons in this study, the range of our $\Phi_{e,C}$ reasonably represented variations in Lake Biwa, including oligotrophic (North Basin) and mesotrophic (South Basin) areas.

The GLM determining $\Phi_{e,C}$ revealed that multiple physicochemical and biological factors, except PAR, were significant in Lake Biwa (Table 6). Previous studies primarily focused on the relationships between light environment, or NPQ_{NSV} and $\Phi_{e,C}$ [25,27,34,38,40,63]. The NPQ_{NSV} is mechanistically linked with alternative electron flow (AEF) activity, which is activated by excess light and photodamage on PSII [1,64,97]. In the present study, PAR intensity was highly correlated with NPQ_{NSV} (Fig. 5), and thus we incorporated PAR in GLM for $\Phi_{e,C}$ as a proxy of excess light and NPQ_{NSV}. Although PAR was not utilized in the development of the best model, water temperature, DO, F_v/F_m,

σ_{PSII}, nutrient conditions, and the compositions of the phytoplankton community were selected. Our results suggest that the light environment is not always the primary factor determining $\Phi_{e,C}$, rather, water temperature plays a critical role in the electron requirement for carbon fixation. Generally, increased temperature decreases the CO_2 affinity of Rubisco through the acceleration of the O_2 evolution rate, and reduction of O_2 solubility [98,99]. Furthermore, increasing temperature may lead to a state of chronic photoinhibition through photodamage [100], as well as changes in species composition of the community [40]. Although we did not examine the interaction among all explanatory variables, temperature may have affected $\Phi_{e,C}$ *vis-à-vis* interaction with the other factors such as nutrient stoichiometry [101].

The relationships between multiple environmental factors other than light and $\Phi_{e,C}$ were reported in previous FRRf [35,36,40] and PAM studies [102,103]. Lawrenz et al. [35] investigated the relationships between the $\Phi_{e,C}$ of marine phytoplankton and environmental conditions, and explored the best fitting models with 14 data sets from various geographic regions. They examined water temperature, salinity, optical depth, attenuation coefficient, Chl-*a*, NO_3, and PO_4 as explanatory variables, and showed only water temperature, NO_3, and PO_4 significantly correlated with $\Phi_{e,C}$ in the data set. Their results support our best model which included water temperature and nutrients.

However, Lawrenz et al. also showed that the coefficients and significance of the identified parameters are quite dependent on region of interest. Thus, future studies are necessary to examine the complex relationship between temperature, light and nutrients conditions in freshwater environments to delineate specific conditions that explain $\Phi_{e,C}$ discrepancies.

Although three excitation wavelength combinations were used to evaluate cyanobacterial photosynthesis correctly, the proportion of cyanobacteria in phytoplankton biomass was significant in determining the best $\Phi_{e,C}$ model (Table 5). The proportion of cyanobacteria within the community may cause differences in the light absorption characteristics between cyanobacteria and the other phytoplankton groups; cyanobacteria absorb only 25–30% of light in PSII, while other phytoplankton absorb 48–58% [42]. Kromkamp et al. (2008) suggested that most of the Chl-*a* in cyanobacteria is associated with PSI rather than PSII, and detection-limited by fluorometry. The FRRf method primarily targets pigments associated with PSII, thus the electron flow initiated by the excited PSI is difficult to evaluate. Therefore, evaluating the proportion of cyanobacteria in phytoplankton biomass might be crucial for correcting GPP estimation with ETR_{PSII} in natural phytoplankton communities.

As expected, PO_4 concentration negatively affected $\Phi_{e,C}$ (Table 5). This result was consistent with previous PO_4 manipulation experiments conducted with marine phytoplankton [104]. The effect of PO_4

was, however, one of the lowest among all factors (Table 5). The low significance is likely due to the fact that the PO_4 concentrations did not change drastically and were generally low (0.01–0.04 μmol L^{-1}, Table 4) throughout the study period.

In order to verify the performance of the $\Phi_{e,C}$ models, we subsequently compared daily GPP estimated with FRRf (GPP_f) and ^{13}C (GPP_{13C}) for the North and the South basins on each sampling date. The RCII-specific primary production based on FRRf (PB_f, mg C mmol RCII $^{-1}$ h^{-1}) was calculated from the J_f, and computed $\Phi_{e,C}$ with the best model, Model 2 and Model 3. The relationships between PB_f and PAR were fitted in *P-E* curves with models [72,84–86], as described in the materials and methods. Finally, daily GPP_f and GPP_{13C} were estimated as follows:

$$GPP = \int_0^Z \{RCII_Z \times \sum_{t=1}^{L} PBi(Z,t)\} \quad (9)$$

where $RCII_Z$ is the RCII concentration at depth Z, L is the day length (h), and $PBi(Z,t)$ is PB_f or PB_{13C} at depth Z and time t (h). The $RCII_Z$ and $PBi(Z,t)$ were calculated every 1.25 m for the North Basin and every 0.5 m for the South Basin based on average values of the observed data. Day length and daily PAR data were obtained from the Japan Meteorological Agency for the North Basin, and were measured by a PAR sensor (PAR-02D; Prede Co., ltd., Tokyo, Japan) at Otsu for the South Basin.

The estimated GPP_{13C} varied from 71 to 787 g C m^{-2} d^{-1}, while GPP_f with best model, the Model

2 and the Model 3 varied from 86 to 630 g C m^{-2} d^{-1}, 85 to 729 g C m^{-2} d^{-1} and 117 to 906 g C m^{-2} d^{-1}, respectively (for date-specific values, see S4 Table). The GPP_f using the best model relative to GPP_{13C} varied from 0.48 to 1.46, suggesting that FRRf parameters with the best $\Phi_{e,C}$ model can reasonably reproduce GPP_{13C} in Lake Biwa (Fig. 6). Relative GPP_f with Model 2 and Model 3, including fewer variables, also replicated GPP_{13C} well, varying from 0.46 to 1.59 and 0.47 to 2.11, respectively. The comparative results suggest that even if nutrients and phytoplankton biomass are not considered, GPP_{13C} can be estimated using the FRRf parameters coupled with temperature for both oligotrophic and mesotrophic area in Lake Biwa. This model parameterization is significant for future applications in freshwater ecosystems where environmental conditions and phytoplankton communities can vary spatially and temporally.

Fig. 6. Relative GPP_f estimated by ETR_{PSII} with the $\Phi_{e,C}$ models against the GPP_{13C} in each sampling date for North and South basins.

In conclusion, the FRRf equipped with excitation wavelengths 633 nm is effective in estimating ETR_{PSII} for freshwater phytoplankton communities during cyanobacterial blooms. Contrary to our

hypothesis, water temperature was the most important determinant factor, while phosphorus concentration was less effective in the $\Phi_{e,C}$ model in Lake Biwa. Further, GPP_{13C} dynamics were effectively estimated from the ETR_{PSII} using the best $\Phi_{e,C}$ model for both oligotrophic and mesotrophic areas in Lake Biwa. The parsimonious model, including only temperature and two photosynthetic parameters also sufficiently reproduced GPP_{13C}. In this study, the phytoplankton community was verified through microscopy, but fluorometric characteristics from the FRRf may also allow researchers to determine phytoplankton assemblages at high *in situ* spatial and temporal resolution [105], and thus simplify the GPP measurements during cyanobacterial blooms. This study provides strong validation for measurements of primary productivity by FRRf in lakes with large spatiotemporal variabilities of phytoplankton assemblages and environmental conditions from oligo- to mesotrophic lakes (or lacustrine) environments. In the future, bio-optical measurements will allow researchers to disentangle the causality between anthropogenic nutrient control and fish catch [13,59,106], and between climate change and the production of higher trophic levels [7,107].

Acknowledgments

We would like to thank Dr. Takamaru Nagata in LBERI and Dr. Hiroki Haga in Lake Biwa

Museum for sharing their ship time and valuable support during sampling. We sincerely thank Dr. Satoshi Nakada and Maho Iwaki for providing PAR profile data, and Hirokazu Teraishi for assisting with the chemical analysis. This study was supported by the Collaborative Research Fund from Shiga Prefecture entitled “Study on water quality and lake-bottom environment for protection of the soundness of water environment” under the Japanese Grant for Regional Revitalization, and the Environment Research and Technology Development Fund (No. 5-1607) of the Ministry of the Environment, Japan.

Author contributions

Conceived and designed the experiments: TK KK. Performed the experiments: TK KH KS. Analyzed the data: TK. Contributed reagents/materials/analysis tools: KH. Supervision: AI. Wrote the original draft: TK. Review and Editing: KH KS VSK AI KK.

Conflict of Interest

None declared.

References

1. Falkowski PG, Raven JA. Aquatic photosynthesis, 2nd edition. New Jersey: Princeton University

Press; 2007. Available: http://site.ebrary.com/id/10789806

2. Oksanen L, Fretwell SD, Arruda J, Niemela P. Exploitation ecosystems in gradients of primary productivity. Am Nat. 1981;118: 240–261. doi:10.1086/283817

3. Power M. Top- Down and Bottom- Up Forces in Food Webs: Do Plants Have Primacy. Ecology. 1992;73: 733–746. doi:10.2307/1940153

4. Falkowski PG, Barber RT, Smetacek V. Biogeochemical controls and feedbacks on ocean primary production. Science. 1998;281: 200. doi:10.1126/science.281.5374.200

5. Field CB. Primary Production of the Biosphere: Integrating Terrestrial and Oceanic Components. Science. 1998;281: 237–240. doi:10.1126/science.281.5374.237

6. Pauly D, Christensen V. Primary production required to sustain global fisheries. Nature. 1995;374: 255.

7. Chassot E, Bonhommeau S, Dulvy NK, Mélin F, Watson R, Gascuel D, et al. Global marine primary production constrains fisheries catches. Ecol Lett. 2010;13: 495–505. doi:10.1111/j.1461-0248.2010.01443.x

8. Hatton IA, McCann KS, Fryxell JM, Davies TJ, Smerlak M, Sinclair ARE, et al. The predator-prey power law: Biomass scaling across terrestrial and aquatic biomes. Science. 2015;349. doi:10.1126/science.aac6284

9. Schindler DW. Factors regulating phytoplankton production and standing crop in the world’s freshwaters: Freshwater phytoplankton production. Limnol Oceanogr. 1978;23: 478–486. doi:10.4319/lo.1978.23.3.0478

10. Carpenter SR, Kitchell JF, Hodgson JR, Cochran PA, Elser JJ, Elser MM, et al. Regulation of lake primary productivity by food web structure. Ecology. 1987;68: 1863–1876. doi:10.2307/1939878

11. Elser JJ, Bracken MES, Cleland EE, Gruner DS, Stanley HW, Helmut H, et al. Global analysis of nitrogen and phosphorus limitation of primary producers in freshwater, marine and terrestrial ecosystems. Ecol Lett. 2007;10: 1135–1142. doi:10.1111/j.1461-0248.2007.01113.x

12. Reynolds CS. The ecology of phytoplankton. Cambridge: Cambridge University Press; 2006.

13. Capuzzo E, Lynam CP, Barry J, Stephens D, Forster RM, Greenwood N, et al. A decline in primary production in the North Sea over 25 years, associated with reductions in zooplankton abundance and fish stock recruitment. Glob Change Biol. 2017 [cited 23 Nov 2017]. doi:10.1111/gcb.13916

14. Nielsen ES. The use of radio-active carbon (C14) for measuring organic production in the sea. ICES J Mar Sci. 1952;18: 117–140. doi:10.1093/icesjms/18.2.117

15. Slawyk G, Collos Y, Auclair J-C. The use of the ^{13}C and ^{15}N isotopes for the simultaneous measurement of carbon and nitrogen turnover rates in marine phytoplankton. Limnol Oceanogr. 1977;22: 925–932. doi:10.4319/lo.1977.22.5.0925

16. Hama T, Miyazaki T, Ogawa Y, Iwakuma T, Takahashi M, Otsuki A, et al. Measurement of photosynthetic production of a marine phytoplankton population using a stable ^{13}C isotope. Mar Biol. 1983;73: 31–36. doi:10.1007/BF00396282

17. Strickland JHD, Parsons TR. A practical handbook of seawater analysis, 2nd edn. Bull Fish Res Board Can. 1972;167: 1–310.

18. Bender ML, Grande KD. Production, respiration, and the isotope geochemistry of O_2 in the upper water column. Glob Biogeochem Cycles. 1987;1: 49–59. doi:10.1029/GB001i001p00049

19. Kolber Z, Falkowski PG. Use of active fluorescence to estimate phytoplankton photosynthesis in situ. Limnol Oceanogr. 1993;38: 1646–1665. doi:10.4319/lo.1993.38.8.1646

20. Kolber ZS, Prášil O, Falkowski PG. Measurements of variable chlorophyll fluorescence using fast repetition rate techniques: defining methodology and experimental protocols. Biochim Biophys Acta BBA - Bioenerg. 1998;1367: 88–106. doi:10.1016/S0005-2728(98)00135-2

21. Suggett D, Kraay G, Holligan P, Davey M, Aiken J, Geider R. Assessment of photosynthesis in a spring cyanobacterial bloom by use of a fast repetition rate fluorometer. Limnol Oceanogr. 2001;46: 802–810. doi:10.4319/lo.2001.46.4.0802

22. Melrose DC, Oviatt CA, O'Reilly JE, Berman MS. Comparisons of fast repetition rate fluorescence estimated primary production and 14C uptake by phytoplankton. Mar Ecol Prog Ser. 2006;311: 37–46.

23. Corno G, Letelier RM, Abbott MR, David MK. Assessing primary production variability in the north pacific subtropical gyre: a comparison of fast repetition rate fluorometry and 14C measurements. J Phycol. 2006;42: 51–60. doi:10.1111/j.1529-8817.2006.00163.x

24. Robinson C, Tilstone GH, Rees AP, Smyth TJ, Fishwick JR, Tarran GA, et al. Comparison of in vitro and in situ plankton production determinations. Aquat Microb Ecol. 2009;54: 13–34.

25. Zhu Y, Ishizaka J, Tripathy SC, Wang S, Mino Y, Matsuno T, et al. Variation of the photosynthetic

electron transfer rate and electron requirement for daily net carbon fixation in Ariake Bay, Japan. J Oceanogr. 2016;72: 761–776. doi:10.1007/s10872-016-0370-4

26. Zhu Y, Ishizaka J, Tripathy S, Wang S, Sukigara C, Goes J, et al. Relationship between light, community composition and the electron requirement for carbon fixation in natural phytoplankton. Mar Ecol Prog Ser. 2017;580: 83–100. doi:10.3354/meps12310

27. Schuback N, Hoppe CJM, Tremblay J-É, Maldonado MT, Tortell PD. Primary productivity and the coupling of photosynthetic electron transport and carbon fixation in the Arctic Ocean. Limnol Oceanogr. 2017;62: 898–921. doi:10.1002/lno.10475

28. Oxborough K, Moore CM, Suggett DJ, Lawson T, Hoi GC, Geider RJ. Direct estimation of functional PSII reaction center concentration and PSII electron flux on a volume basis: a new approach to the analysis of Fast Repetition Rate fluorometry (FRRf) data. Limnol Oceanogr Methods. 2012;10: 142–154. doi:10.4319/lom.2012.10.142

29. Smyth TJ, Pemberton KL, Aiken J, Geider RJ. A methodology to determine primary production and phytoplankton photosynthetic parameters from Fast Repetition Rate Fluorometry. J Plankton Res. 2004;26: 1337–1350. doi:10.1093/plankt/fbh124

30. Tripathy SC, Ishizaka J, Fujiki T, Shibata T, Okamura K, Hosaka T, et al. Assessment of carbon- and fluorescence-based primary productivity in Ariake Bay, southwestern Japan. Estuar Coast Shelf Sci. 2010;87: 163–173. doi:10.1016/j.ecss.2010.01.006

31. Komatsu K, Imai A, Tomioka N, Takamura N, Nakagawa M, Satou T, et al. Method development for determining primary production rate by fast repetition rate fluorometry (FRRF). J Jpn Soc Civ Eng Ser G Environ Res. 2015;71: III_309-III_314. doi:10.2208/jscejer.71.III_309

32. Regaudie-de-Gioux A, Lasternas S, Agustí S, Duarte CM. Comparing marine primary production estimates through different methods and development of conversion equations. Front Mar Sci. 2014;1: 19. doi:10.3389/fmars.2014.00019

33. Hughes D, Campbell D, Doblin MA, Kromkamp J, Lawrenz E, Moore CM, et al. Roadmaps and detours: active chlorophyll-a assessments of primary productivity across marine and freshwater systems. Environ Sci Technol. 2018. doi:10.1021/acs.est.8b03488

34. Schuback N, Flecken M, Maldonado MT, Tortell PD. Diurnal variation in the coupling of photosynthetic electron transport and carbon fixation in iron-limited phytoplankton in the NE subarctic Pacific. Biogeosciences. 2016;13: 1019–1035. doi:10.5194/bg-13-1019-2016

35. Lawrenz E, Silsbe G, Capuzzo E, Ylöstalo P, Forster RM, Simis SGH, et al. Predicting the electron requirement for carbon fixation in seas and oceans. Stal LJ, editor. PLoS ONE. 2013;8: e58137. doi:10.1371/journal.pone.0058137

36. Robinson C, Suggett DJ, Cherukuru N, Ralph PJ, Doblin MA. Performance of fast repetition rate fluorometry based estimates of primary productivity in coastal waters. J Mar Syst. 2014;139: 299–310. doi:10.1016/j.jmarsys.2014.07.016

37. Silsbe GM, Smith REH, Twiss MR. Quantum efficiency of phytoplankton photochemistry measured continuously across gradients of nutrients and biomass in Lake Erie (Canada and USA) is strongly regulated by light but not by nutrient deficiency. Can J Fish Aquat Sci. 2015;72: 651–660. doi:10.1139/cjfas-2014-0365

38. Schuback N, Schallenberg C, Duckham C, Maldonado MT, Tortell PD. Interacting effects of light and iron availability on the coupling of photosynthetic electron transport and CO_2-assimilation in marine phytoplankton. Cockshutt AM, editor. PLOS ONE. 2015;10: e0133235. doi:10.1371/journal.pone.0133235

39. Hughes DJ, Varkey D, Doblin MA, Ingleton T, Mcinnes A, Ralph PJ, et al. Impact of nitrogen

availability upon the electron requirement for carbon fixation in Australian coastal phytoplankton communities. Limnol Oceanogr. 2018;63: 1891–1910. doi:10.1002/lno.10814

40. Ryan-Keogh TJ, Thomalla SJ, Little H, Melanson J-R. Seasonal regulation of the coupling between photosynthetic electron transport and carbon fixation in the Southern Ocean. Limnol Oceanogr. 2018;63: 1856–1876. doi:10.1002/lno.10812

41. Raateoja M, Seppälä J, Ylöstalo P. Fast repetition rate fluorometry is not applicable to studies of filamentous cyanobacteria from the Baltic Sea. Limnol Oceanogr. 2004;49: 1006–1012. doi:10.4319/lo.2004.49.4.1006

42. Suggett DJ, MacIntyre HL, Geider RJ. Evaluation of biophysical and optical determinations of light absorption by photosystem II in phytoplankton: Evaluation of light absorption by PSII. Limnol Oceanogr Methods. 2004;2: 316–332. doi:10.4319/lom.2004.2.316

43. Suggett D, MacIntyre H, Kana T, Geider R. Comparing electron transport with gas exchange: parameterising exchange rates between alternative photosynthetic currencies for eukaryotic phytoplankton. Aquat Microb Ecol. 2009;56: 147–162. doi:10.3354/ame01303

44. Kulk G, van de Poll WH, Buma AGJ. Photophysiology of nitrate limited phytoplankton

communities in Kongsfjorden, Spitsbergen: Photophysiology of Arctic phytoplankton. Limnol Oceanogr. 2018 [cited 30 Aug 2018]. doi:10.1002/lno.10963

45. Roach T, Krieger-Liszkay AK. Regulation of photosynthetic electron transport and photoinhibition. Curr Protein Pept Sci. 2014;15: 351–362. doi:10.2174/1389203715666140327105143

46. Oliver RL, Ganf GG. Freshwater Blooms. In: Whitton BA, Potts M, editors. The Ecology of Cyanobacteria: Their Diversity in Time and Space. Dordrecht: Springer; 2002. pp. 149–194. doi:10.1007/0-306-46855-7_6

47. Paerl HW, Scott JT, McCarthy MJ, Newell SE, Gardner WS, Havens KE, et al. It takes two to tango: when and where dual nutrient (N & P) reductions are needed to protect lakes and downstream ecosystems. Environ Sci Technol. 2016;50: 10805–10813. doi:10.1021/acs.est.6b02575

48. Kaiblinger C, Dokulil MT. Application of fast repetition rate fluorometry to phytoplankton photosynthetic parameters in freshwaters. Photosynth Res. 2006;88: 19–30. doi:10.1007/s11120-005-9018-8

49. Wojtasiewicz B, Stoń-Egiert J. Bio-optical characterization of selected cyanobacteria strains present in marine and freshwater ecosystems. J Appl Phycol. 2016;28: 2299–2314. doi:10.1007/s10811-015-0774-3

50. Houliez E, Simis S, Nenonen S, Ylöstalo P, Seppälä J. Basin-scale spatio-temporal variability and control of phytoplankton photosynthesis in the Baltic Sea: The first multiwavelength fast repetition rate fluorescence study operated on a ship-of-opportunity. J Mar Syst. 2017;169: 40–51. doi:10.1016/j.jmarsys.2017.01.007

51. Conley DJ, Paerl HW, Howarth RW, Boesch DF, Seitzinger SP, Havens KE, et al. Ecology: controlling eutrophication: nitrogen and phosphorus. Science. 2009;323: 1014–1015. doi:10.1126/science.1167755

52. Karlsson J, Byström P, Ask J, Ask P, Persson L, Jansson M. Light limitation of nutrient-poor lake ecosystems. Nature. 2009;460: 506–509. doi:10.1038/nature08179

53. Howarth RW. Nutrient limitation of net primary production in marine ecosystems. Annu Rev Ecol Syst. 1988;19: 89–110.

54. Howarth RW, Marino R. Nitrogen as the limiting nutrient for eutrophication in coastal marine

ecosystems: Evolving views over three decades. Limnol Oceanogr. 2006;51: 364–376. doi:10.4319/lo.2006.51.1_part_2.0364

55. Sterner RW, Elser JJ. Ecological stoichiometry: the biology of elements from molecules to the biosphere. Princeton, New Jersey, USA: Princeton University Press; 2002.

56. Pemberton KL, Smith RE, Silsbe GM, Howell T, Watson SB. Controls on phytoplankton physiology in Lake Ontario during the late summer: evidence from new fluorescence methods. Can J Fish Aquat Sci. 2007;64: 58–73. doi:10.1139/f06-166

57. Kaiblinger C, Greisberger S, Teubner K, Dokulil MT. Photosynthetic efficiency as a function of thermal stratification and phytoplankton size structure in an oligotrophic alpine lake. Hydrobiologia. 2007;578: 29–36. doi:10.1007/s10750-006-0430-7

58. Kromkamp JC, Dijkman NA, Peene J, Simis SGH, Gons HJ. Estimating phytoplankton primary production in Lake IJsselmeer (The Netherlands) using variable fluorescence (PAM-FRRF) and C-uptake techniques. Eur J Phycol. 2008;43: 327–344. doi:10.1080/09670260802080895

59. Kawanabe H, Nishino M, Maehata M, editors. Lake Biwa: interactions between nature and people. Dordrecht ; New York ; London: Springer; 2012.

60. Nishino M. Ecological Changes in Lake Biwa. In: Kawanabe H, Nishino M, Maehata M, editors. Lake Biwa: Interactions between Nature and People. Dordrecht: Springer; 2012. pp. 155–238. doi:10.1007/978-94-007-1783-1_3

61. Suzuki R, Ishimaru T. An improved method for the determination of phytoplankton chlorophyll using N, N-dimethylformamide. J Oceanogr Soc Jpn. 1990;46: 190–194. doi:10.1007/BF02125580

62. Boatman TG, Geider RJ, Oxborough K. Improving the accuracy of single turnover active fluorometry (STAF) for the estimation of phytoplankton primary productivity (phytoPP). Front Mar Sci. 2019;6. doi:10.3389/fmars.2019.00319

63. Wei Y, Zhao X, Sun J, Liu H. Fast repetition rate fluorometry (FRRF) derived phytoplankton primary productivity in the Bay of Bengal. Front Microbiol. 2019;10. doi:10.3389/fmicb.2019.01164

64. Nawrocki WJ, Tourasse NJ, Taly A, Rappaport F, Wollman F-A. The plastid terminal oxidase: its elusive function points to multiple contributions to plastid physiology. Annu Rev Plant Biol. 2015;66: 49–74. doi:10.1146/annurev-arplant-043014-114744

65. McKew BA, Davey P, Finch SJ, Hopkins J, Lefebvre SC, Metodiev MV, et al. The trade-off between the light-harvesting and photoprotective functions of fucoxanthin-chlorophyll proteins dominates light acclimation in Emiliania huxleyi (clone CCMP 1516). New Phytol. 2013;200: 74–85. doi:10.1111/nph.12373

66. Hoppe CJM, Holtz L-M, Trimborn S, Rost B. Ocean acidification decreases the light-use efficiency in an Antarctic diatom under dynamic but not constant light. New Phytol. 2015;207: 159–171. doi:10.1111/nph.13334

67. Kitajima M, Butler WL. Quenching of chlorophyll fluorescence and primary photochemistry in chloroplasts by dibromothymoquinone. Biochim Biophys Acta. 1975;376: 105–115. doi:10.1016/0005-2728(75)90209-1

68. Wetzel RG, Likens GE. Limnological analyses. New York, NY: Springer; 2010. Available: http://dx.doi.org/10.1007/978-1-4757-3250-4

69. Luz B, Barkan E, Sagi Y, Yacobi YZ. Evaluation of community respiratory mechanisms with oxygen isotopes: A case study in Lake Kinneret. Limnol Oceanogr. 2002;47: 33–42. doi:10.4319/lo.2002.47.1.0033

70. Pringault O, Tassas V, Rochelle-Newall E. Consequences of respiration in the light on the determination of production in pelagic systems. Biogeosciences. 2007;4: 105–114.

71. Sorokin YI. The Radiocarbon Method to Estimate Primary Production in Aquatic Environments. In: Sorokin YI, editor. Radioisotopic Methods in Hydrobiology. Berlin, Heidelberg: Springer; 1999. pp. 21–99. doi:10.1007/978-3-642-59934-7_2

72. Webb WL, Newton M, Starr D. Carbon dioxide exchange of Alnus rubra. Oecologia. 1974;17: 281–291. doi:10.1007/BF00345747

73. Hillebrand H, Dürselen C-D, Kirschtel D, Pollingher U, Zohary T. Biovolume calculation for pelagic and benthic microalgae. J Phycol. 1999;35: 403–424. doi:10.1046/j.1529-8817.1999.3520403.x

74. Menden-Deuer S, Lessard EJ. Carbon to volume relationships for dinoflagellates, diatoms and other protist plankton. Limnol Oceanogr. 2000;45: 569–579. doi:10.4319/lo.2000.45.3.0569

75. Miyajima T, Yamada Y, Wada E, Nakajima T, Koitabashi T, Hanba YT, et al. Distribution of greenhouse gases, nitrite, and $\delta^{13}C$ of dissolved inorganic carbon in Lake Biwa: Implications for hypolimnetic metabolism. Biogeochemistry. 1997;36: 205–221. doi:10.1023/A:1005702707183

76. Belzile C, Vincent WF, Howard- Williams C, Hawes I, James MR, Kumagai M, et al. Relationships between spectral optical properties and optically active substances in a clear oligotrophic lake. Water Resour Res. 2004;40. doi:10.1029/2004WR003090

77. Kirk JTO. Light and photosynthesis in aquatic ecosystems. 3rd ed. Cambridge, UK ; New York: Cambridge University Press; 2011.

78. Paavel B, Kangro K, Arst H, Reinart A, Kutser T, Nõges T. Parameterization of chlorophyll-specific phytoplankton absorption coefficients for productive lake waters. J Limnol. 2016;75. doi:10.4081/jlimnol.2016.1426

79. Ylöstalo P, Kallio K, Seppälä J. Absorption properties of in-water constituents and their variation among various lake types in the boreal region. Remote Sens Environ. 2014;148: 190–205. doi:10.1016/j.rse.2014.03.023

80. Pope RM, Fry ES. Absorption spectrum (380–700 nm) of pure water. II. Integrating cavity measurements. Appl Opt. 1997;36: 8710–8723. doi:10.1364/AO.36.008710

81. Belzile C, Vincent WF, Kumagai M. Contribution of absorption and scattering to the attenuation of UV and photosynthetically available radiation in Lake Biwa. Limnol Oceanogr. 2002;47: 95–

107. doi:10.4319/lo.2002.47.1.0095

82. Terrel MM, Fukushima T, Matsushita B, Yoshimura K, Imai A. Long-term light environment variability in Lake Biwa and Lake Kasumigaura, Japan: modeling approach. Limnology. 2012;13: 237–252. doi:10.1007/s10201-012-0372-x

83. New Energy and Industrial Technology Development Organization (NEDO). Solar radiation spectrum database. 2016. Available: http://app0_2.infoc.nedo.go.jp/

84. Peeters JCH, Eilers P. The relationship between light intensity and photosynthesis—A simple mathematical model. Hydrobiol Bull. 1978;12: 134–136. doi:10.1007/BF02260714

85. Aiba S. Growth kinetics of photosynthetic microorganisms. Microbial Reactions. 1982. pp. 85–156.

86. Megard RO, Tonkyn DW, Senft WH II. Kinetics of oxygenic photosynthesis in planktonic algae. J Plankton Res. 1984;6: 325–337. doi:10.1093/plankt/6.2.325

87. R Development Core Team. R : A Language and environment for statistical computing. 2017. Available: http//www.R-project.org/

88. O'brien RM. A caution regarding rules of thumb for variance inflation factors. Qual Quant. 2007;41: 673–690. doi:10.1007/s11135-006-9018-6

89. Fox J, Weisberg S, Price B, Adler D, Bates D, Baud-Bovy G, et al. car: Companion to Applied Regression. 2019. Available: https://CRAN.R-project.org/package=car

90. Barton K. MuMIn: multi-model inference v1.15.1. 2015. Available: https://cran.r-project.org/web/packages/MuMIn/index.html

91. Akaike H. Information theory and an extension of the maximum likelihood principle. 2nd Int Symp Inf Theory. 1973; 267–281.

92. Johnsen G, Sakshaug E. Biooptical characteristics of PSII and PSI in 33 species (13 pigment groups) of marine phytoplankton, and the relevance for pulse-amplitude-modulated and fast-repetition-rate fluorometry. J Phycol. 2007;43: 1236–1251. doi:10.1111/j.1529-8817.2007.00422.x

93. Suggett DJ, Moore CM, Hickman AE, Geider RJ. Interpretation of fast repetition rate (FRR) fluorescence: signatures of phytoplankton community structure versus physiological state. Mar Ecol Prog Ser. 2009;376: 1–19. doi:10.3354/meps07830

94. Simis SGH, Huot Y, Babin M, Seppälä J, Metsamaa L. Optimization of variable fluorescence measurements of phytoplankton communities with cyanobacteria. Photosynth Res. 2012;112: 13–30. doi:10.1007/s11120-012-9729-6

95. Bricaud A, Babin M, Morel A, Claustre H. Variability in the chlorophyll-specific absorption coefficients of natural phytoplankton: Analysis and parameterization. J Geophys Res Oceans. 1995;100: 13321–13332. doi:10.1029/95JC00463

96. Stæhr PA, Markager S. Parameterization of the chlorophyll a-specific in vivo light absorption coefficient covering estuarine, coastal and oceanic waters. Int J Remote Sens. 2004;25: 5117–5130. doi:10.1080/01431160410001716932

97. Saroussi SI, Wittkopp TM, Grossman AR. The type II NADPH dehydrogenase facilitates cyclic electron flow, energy-dependent quenching, and chlororespiratory metabolism during acclimation of chlamydomonas reinhardtii to nitrogen deprivation. Plant Physiol. 2016;170: 1975. doi:10.1104/pp.15.02014

98. Ogren WL. Photorespiration:pathways, regulation, and modification. Annu Physiol. 1984;35: 415–442. doi:10.1146/annurev.pp.35.060184.002215

99. Hagemann M, Weber AP, Eisenhut M. Photorespiration: origins and metabolic integration in interacting compartments. J Exp Bot. 2016;67: 2915–2918. doi:10.1093/jxb/erw178

100. Yellowlees D, Warner M. Photosynthesis in Symbiotic Algae. In: Larkum AWD, Douglas SE, Raven JA, editors. Photosynthesis in Algae. Dordrecht: Springer Netherlands; 2003. pp. 437–455. doi:10.1007/978-94-007-1038-2_19

101. Fernández‐ González C, Pérez‐ Lorenzo M, Pratt N, Moore CM, Bibby TS, Marañón E. Effects of Temperature and Nutrient Supply on Resource Allocation, Photosynthetic Strategy, and Metabolic Rates of Synechococcus sp. J Phycol. 2020;56: 818–829. doi:10.1111/jpy.12983

102. Napoléon C, Claquin P. Multi-parametric relationships between PAM measurements and carbon incorporation, an in situ approach. PLOS ONE. 2012;7: e40284. doi:10.1371/journal.pone.0040284

103. Morelle J, Schapira M, Orvain F, Riou P, Lopez PJ, Pierre-Duplessix O, et al. Annual phytoplankton primary production estimation in a temperate estuary by coupling pam and carbon incorporation methods. Estuaries Coasts. 2018;41: 1337–1355. doi:10.1007/s12237-018-0369-8

104. Napoléon C, Raimbault V, Claquin P. Influence of nutrient stress on the relationships between

PAM measurements and carbon incorporation in four phytoplankton species. PLOS ONE. 2013;8: e66423. doi:10.1371/journal.pone.0066423

105. Rolland A, Rimet F, Jacquet S. A 2-year survey of phytoplankton in the Marne Reservoir (France): A case study to validate the use of an in situ spectrofluorometer by comparison with algal taxonomy and chlorophyll a measurements. Knowl Manag Aquat Ecosyst. 2010; 02. doi:10.1051/kmae/2010023

106. Yanagi T. Oligotrophication in the Seto Inland Sea. In: Yanagi T, editor. Eutrophication and Oligotrophication in Japanese Estuaries: The present status and future tasks. Dordrecht: Springer; 2015. pp. 39–67. doi:10.1007/978-94-017-9915-7_3

107. Ware DM, Thomson RE. Bottom-up ecosystem trophic dynamics determine fish production in the Northeast Pacific. Science. 2005;308: 1280–1284. doi:10.1126/science.1109049

S1 Table. Spectral correction factor (SFC) for JV_f estimation in each sampling date.

S2 Table. All GLM submodels with standardization of variables.

S3 Table. All GLM submodels without standardization of variables

S4 Table. GPP (g C m^{-2} d^{-1}) estimated by ^{13}C and FRRf with $\Phi_{e,C}$ models in each sampling date.

S1 Appendix. Spectral distribution of (A) excitation flash of FastOcean (B) light source of growth-chamber.

S2 Appendix. Modeled absorption spectrum for various Chl-*a* concentration. The spectrum were calculated with Paavel's model [78] for 30 and 40 μg L^{-1} in August, and Ylöstalo's model [79] for others.

S3 Appendix. Modeled absorption spectrum of (A) pure water, (B) CDOM and (C) non-algal particles.

S4 Appendix. Spectral distribution of incident sunlight at 10:00 in (A) April to September, (B) October to February and (C) at 4 PM in July in 2015. Each spectrum distribution was referred to calculate the spectral correction in each sampling date as in equation (7).

S5 Appendix. Relative contribution to total phytoplankton biomass by algal groups on each sampling date.

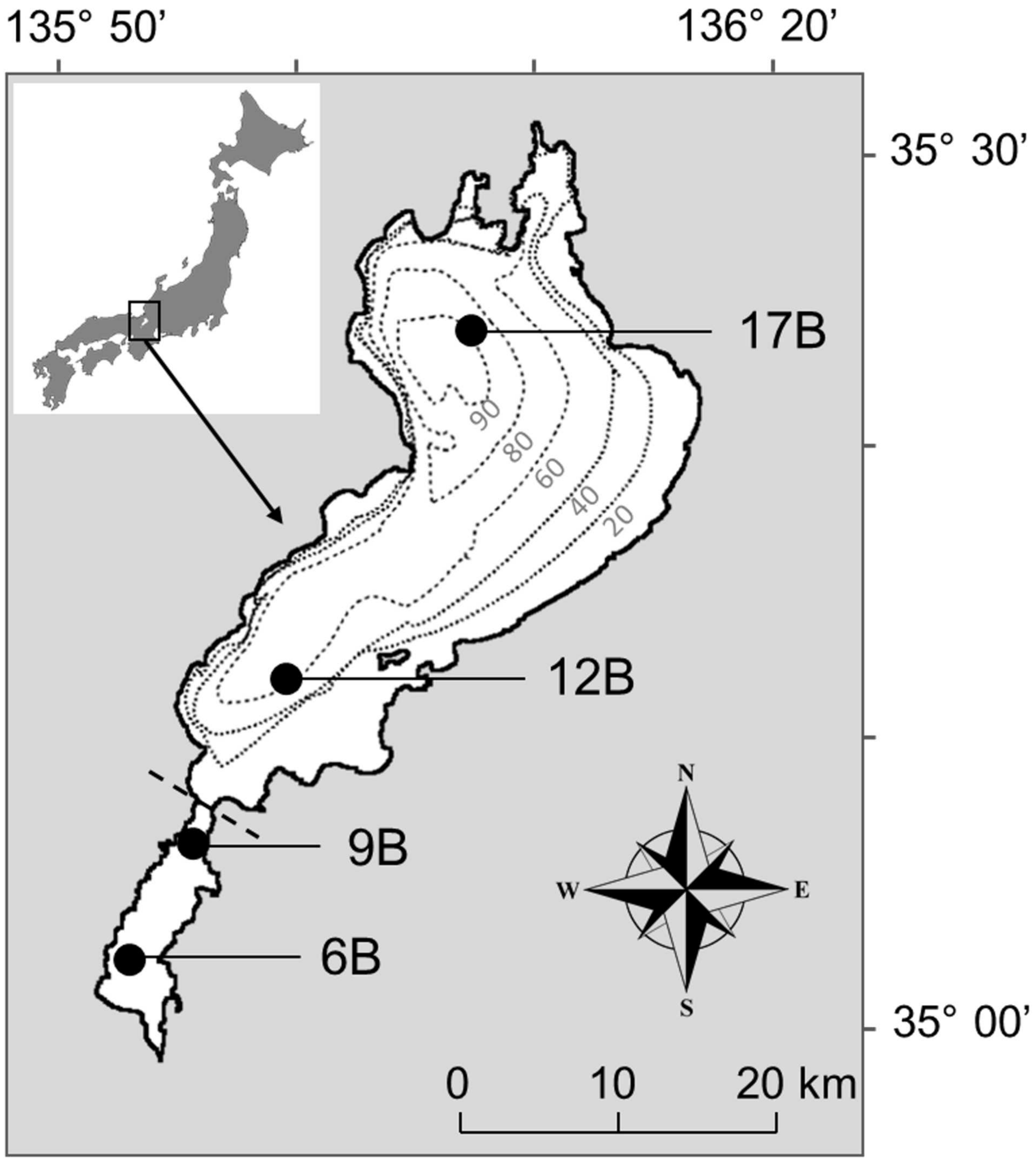
135° 50'
136° 20'
35° 30'
35° 00'
17B
12B
9B
6B
90
80
60
40
20
N
W
E
S
0
10
20 km

 Click here to access/download;Figure;Fig2.eps

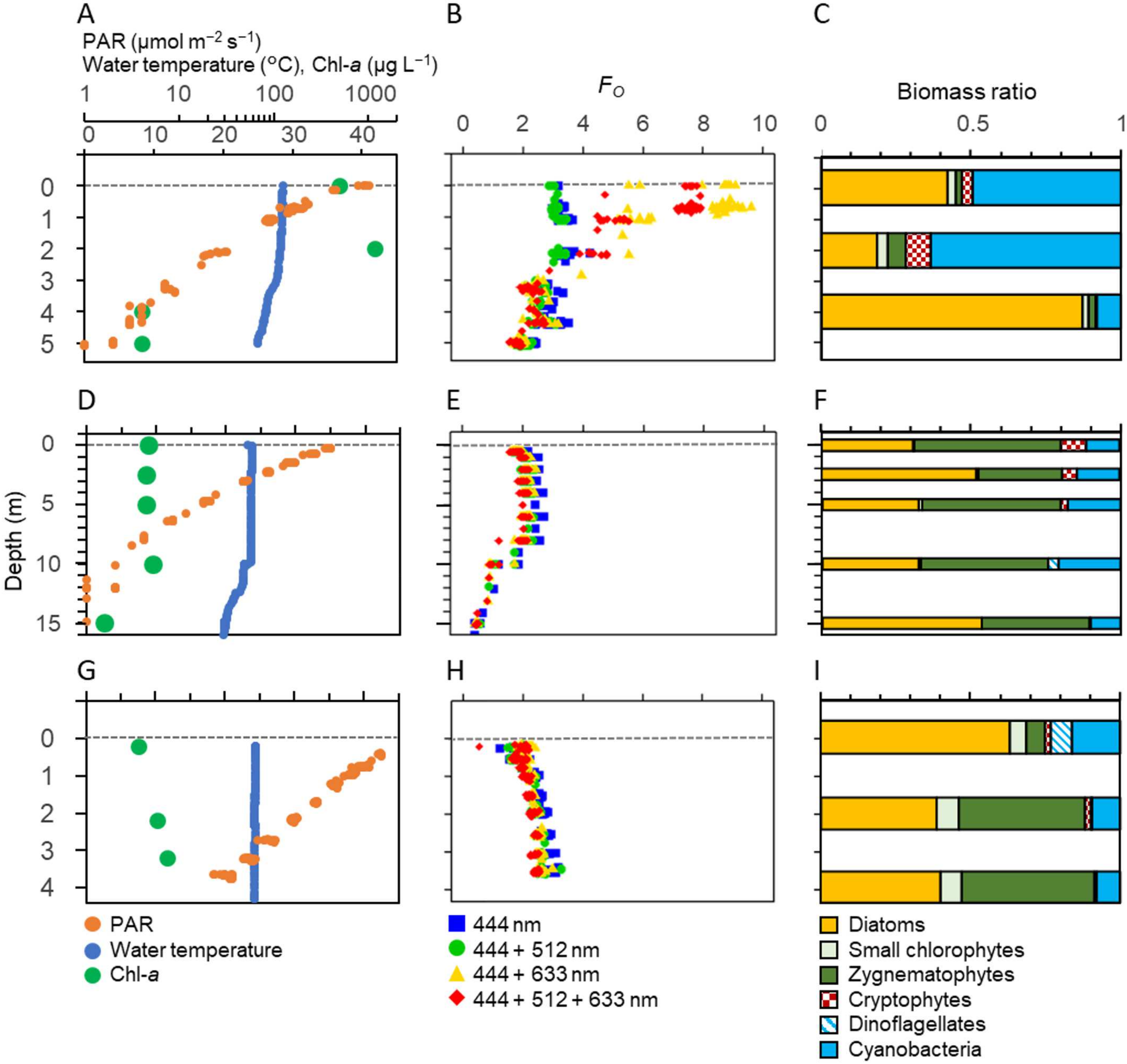

Figure3

Click here to access/download;Figure;Fig3.eps

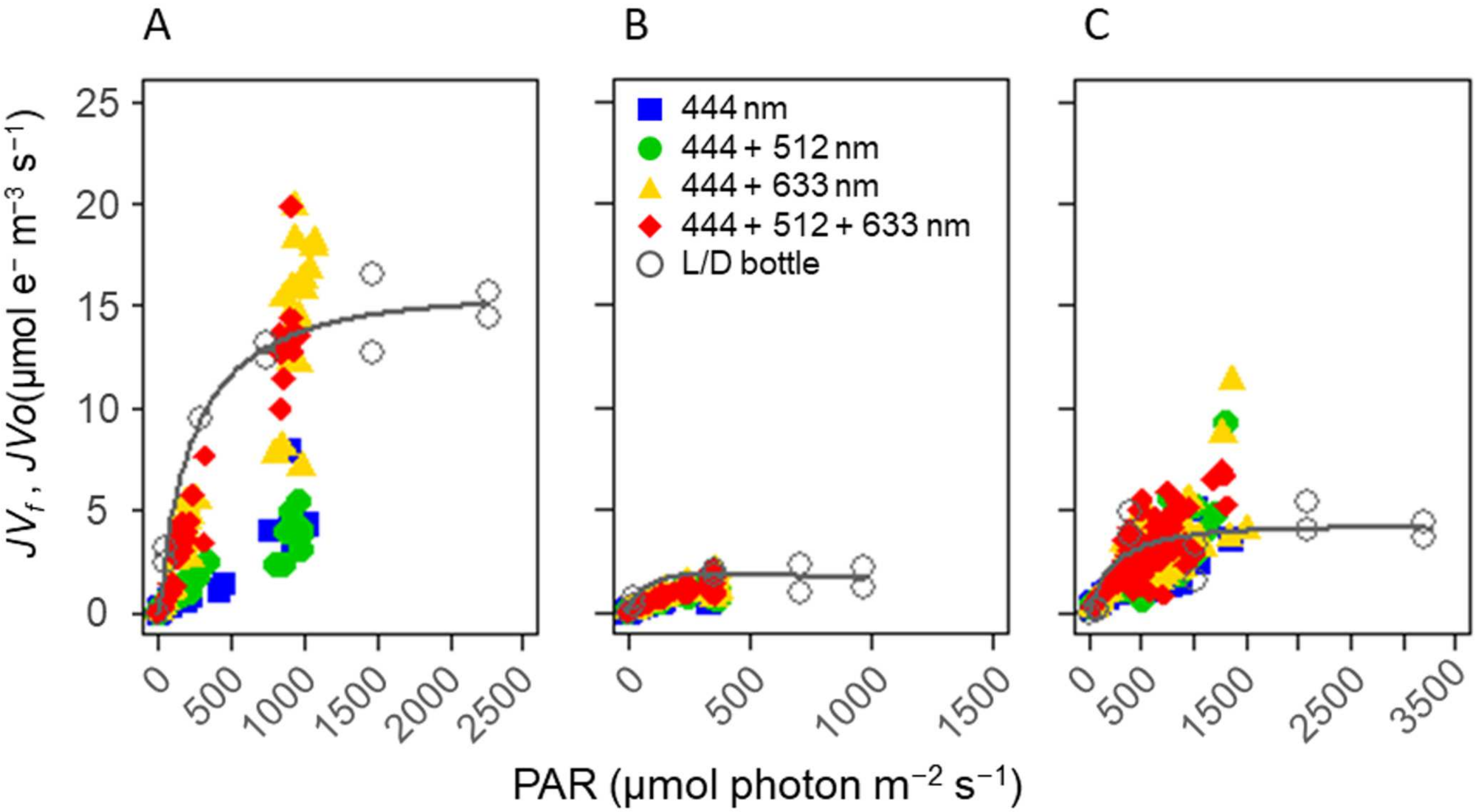

Figure4

Click here to access/download;Figure;Fig4.eps

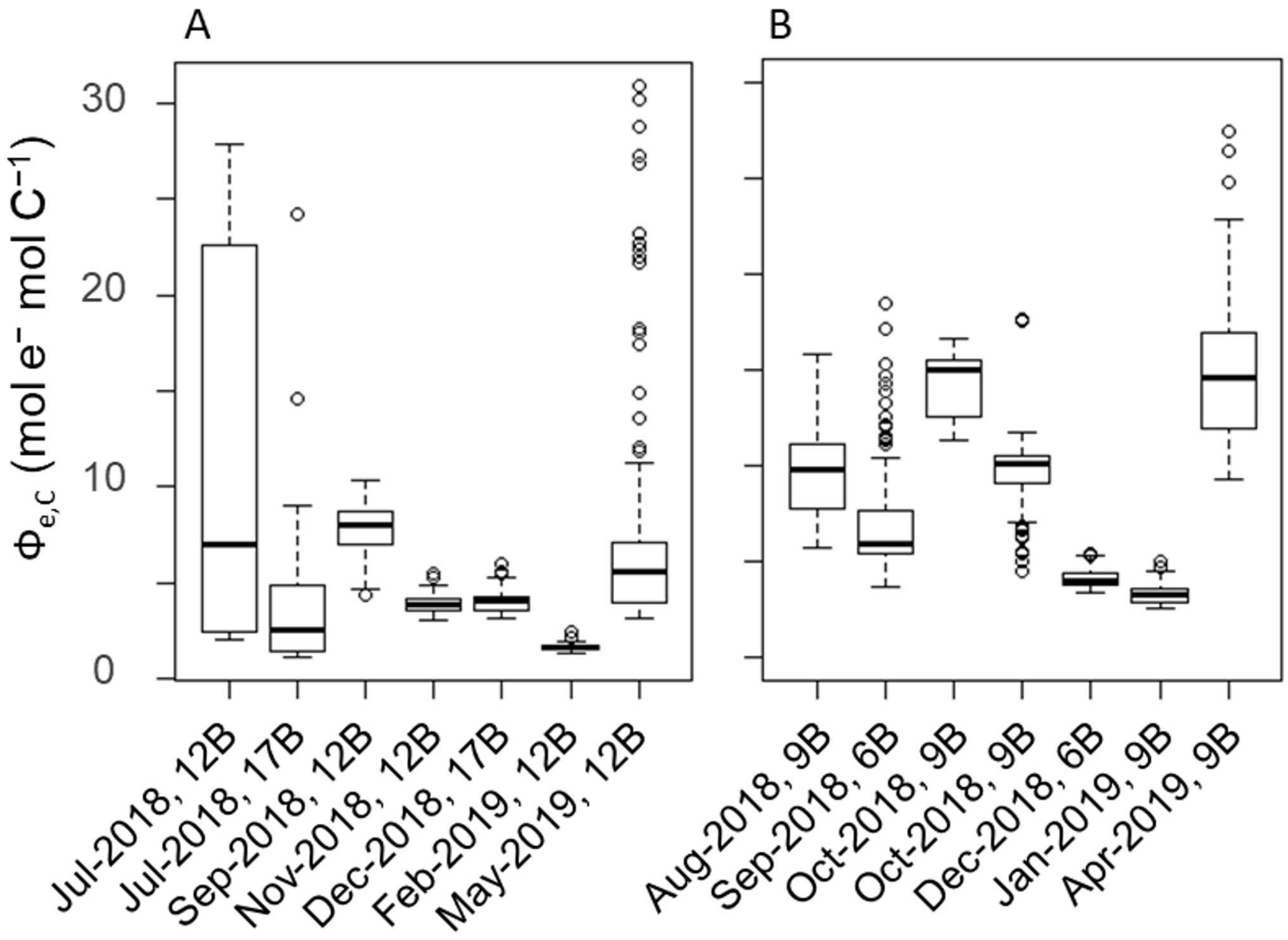

Figure5 Click here to access/download;Figure;Fig5.eps

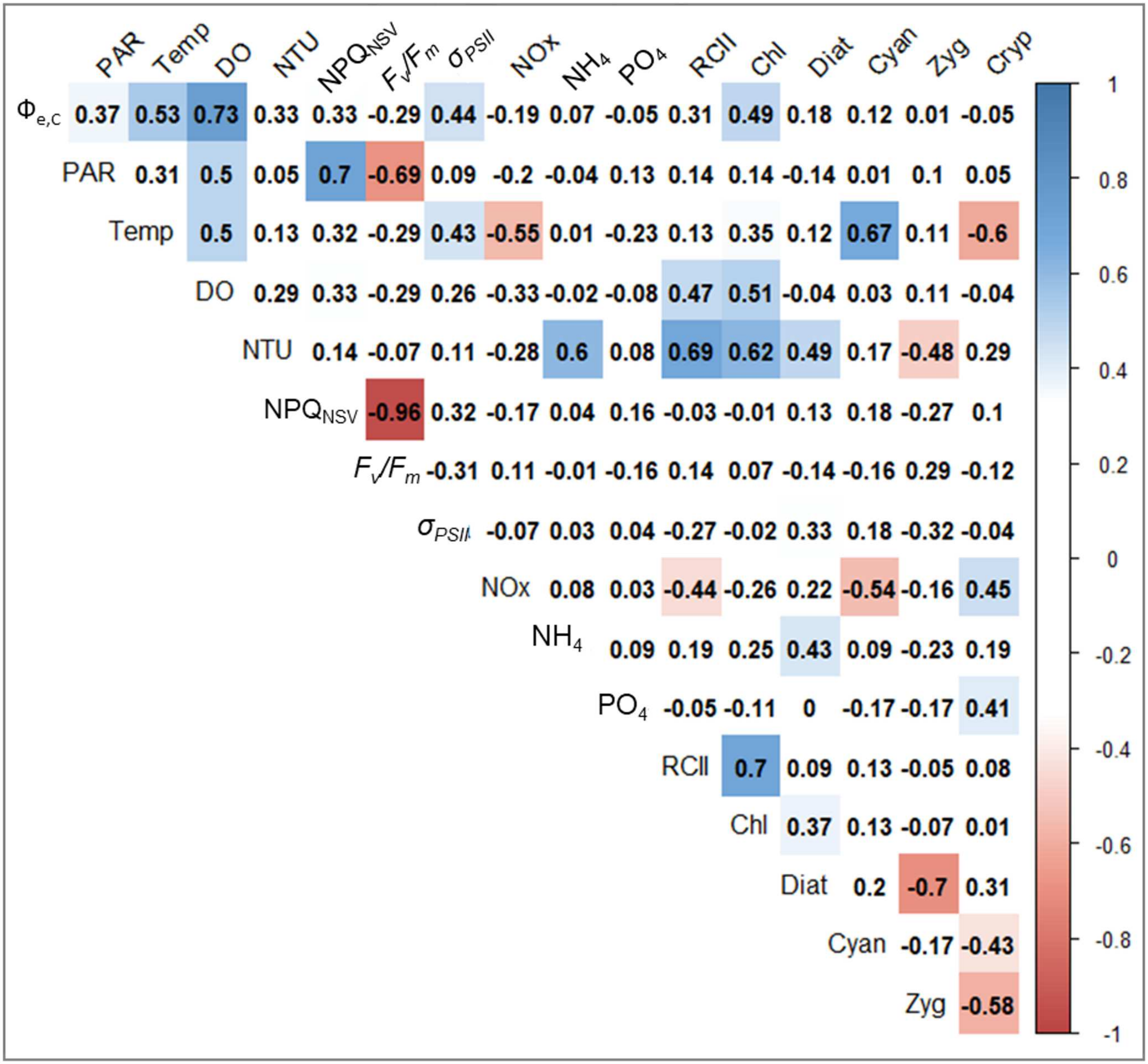

 Click here to access/download;Figure;Fig6.eps

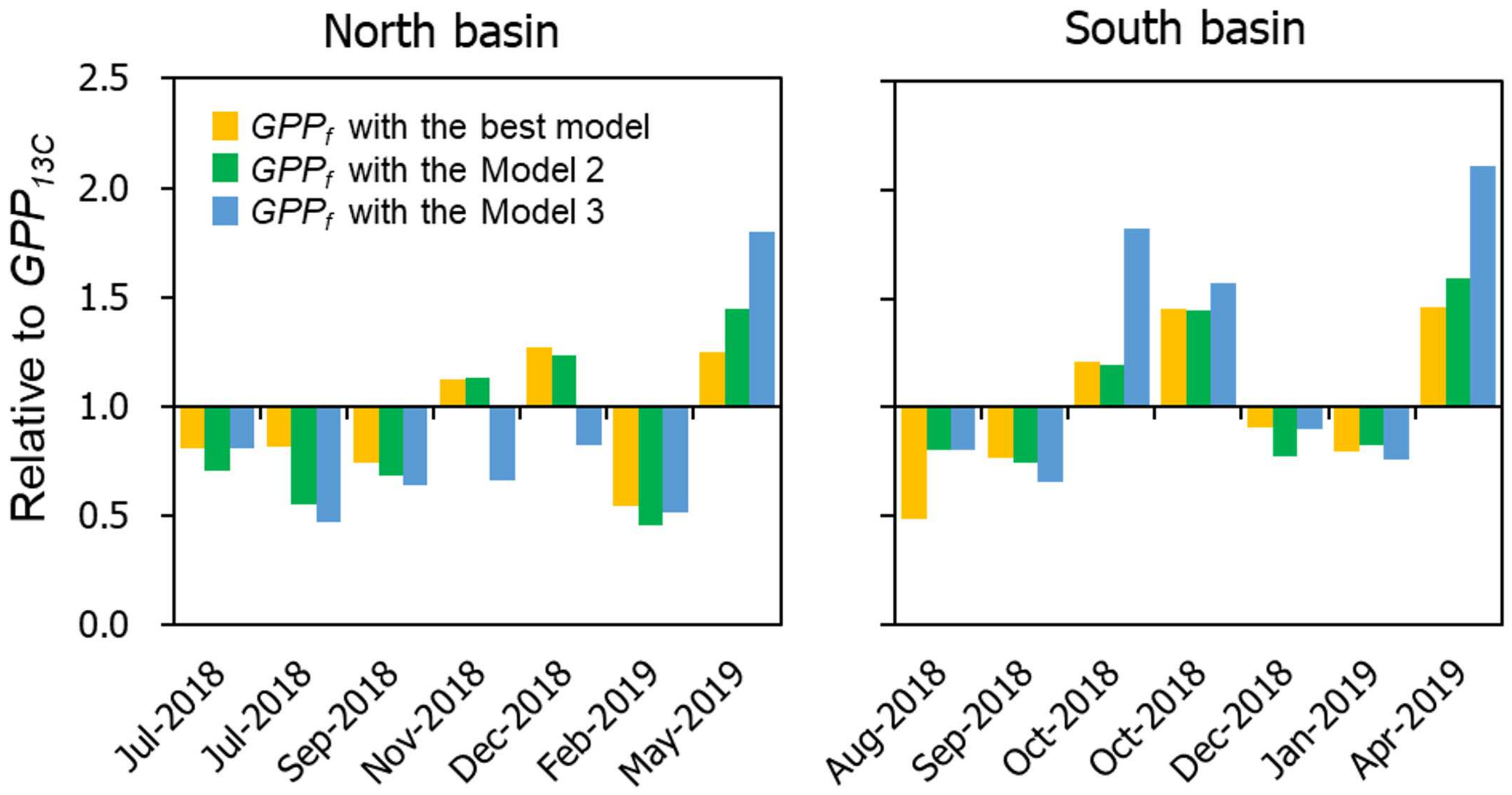

S1Table

Click here to access/download
Supporting Information
S1Table.docx

S2 Table

Click here to access/download
Supporting Information
S2Table.csv

S3 Table

Click here to access/download
Supporting Information
S3Table.csv

S4Table

Click here to access/download
Supporting Information
S4Table.docx
