# Supplementary material for: Development of photosynthetic carbon fixation model using multi-excitation wavelength fast repetition rate fluorometry in Lake Biwa
Source: PLoS One. 2021 Feb 2;16(2):e0238013. doi: 10.1371/journal.pone.0238013 (PMC7853527; doi:10.1371/journal.pone.0238013)
Supplement: Attachment — Submitted filename: response to reviewers3_TK0106_TK.docx [file pone.0238013.s013.docx]

Editor comments

Personally, I would like to see some of the other reviewers original comments addressed in the manuscript. Namely:

Reviewer 2

The comment on L79-81 of the original manuscript was answered on your review but nothing was added to the manuscript, please do so. The same for L104-105 comment.

>>> We revised the manuscript to match our reply as follows,

“Light availability can be affected by the concentrations of colored dissolved organic matter (CDOM), non-algal particles, and phytoplankton. Moreover, the vertical distribution of phytoplankton is markedly different in open-ocean and coastal areas, where the former has peak biomass located in a much deeper area with less CDOM and non-algal particles in the water column, allowing more light availability [35].” (L**79-83**)

>>> L104-105 was revised as follows,

“Whereas nitrogen limitation depresses cellular Chl-a concentration, phosphorus limitation inhibits RNA and ATP synthesis, which can affect protein synthesis and photochemical energy conversion in algae [56, 57].” (L**110-111**)

Reviewer 3

Your answer to the first comment merits being added to the M&M.

>>> We included the following to the M&M in L**343-349**.

“In Lake Biwa, Anabaena affinis and Aphanothece sp. dominated >90% of the cyanobacterial biomass at 0 and 2 m, respectively, in August 2018. The former species has phycocyanin (PC) [49], whereas the latter has PC and phycoerythrin (PE) [88,89]. Paavel et al. [86] described a summer cyanobacterial bloom that consisted of Anabaena, Aphanizomenon, and Gloeotrichia. The former two species have PC [49], and later species has PC and PE [90]. We tested the a_phy^* (λ) of pure Anabaena [49] culture in August 2018 in Lake Biwa, but the SCF changed to only 3%. We believe that Paavel et al. a_phy^* (λ) coefficients are better for the abovementioned reason.”

>>>In addition, we checked all manuscript through English editing service, and fixed following words.

analyser → analyzer (L**176&320**)

categorised → categorized (L**291**)

Reviewer #1: The author's responses have largely addressed my comments and concerns.

I remain concerned about predictive modelling based upon impossible data. Unless something deeply unexpected is happening phi e, C cannot be below 4.

Yet many/most of the estimated values for phi e, C are below 4.

So, I think it should be crystal clear, throughout, that the authors have applied relatively standard approaches, and, consistent with previous attempts, are getting mechanistically impossible answers. So, the field is, consistently, generating paradoxical findings which most likely reflect core issues with the approaches.

>>> Thank you for pointing this out. We added the some notices about unrealistic Фe,C values (<4) in abstract and discussion as follows,

“This study quantifies the in situ FRRf method in a freshwater ecosystem, discusses core issues in the methodology to calculate Фe,C, and assesses the applicability of the method for lake GPP prediction.”(L**41-42**)

“It is also possible that the geometric properties of the incubation vessel can alter the light environment on each bottle, and cause calculation errors in ETRPSII and underestimation of Фe,C [43]. In any case, although we applied relatively standard methods throughout the study, i.e., FRRf vs. bottle incubations with an isotopic tracer, the results showed Фe,C < 4, which may reflect core issues in this approach.

The Фe,C values < 4 have often been observed in laboratory cultures [43,70], natural communities in open oceans [26,40,44], and coastal regions [25,36,39]. However, the analysis method for the low Фe,C values is not yet unified. Most of the studies included the low Фe,C values in the statistical analyses [36,39,40,44,70], while a few studies did not [25,26]. In this study, we regarded all of the Фe,C values as apparent values as in the previous study [35] since the bottle incubation method can cause artifacts for all bottles. Thus, the low Фe,C values were included in the GLM analysis to clarify the apparent effect of environmental and biological factors on the variance of Фe,C.” (**L575-586**)

“These results must be interpreted with caution because although Фe,C should be mechanistically ≥ 4 mol e− mol C−1, our study showed Фe,C < 4 in 9 out of 14 observations. In this study, we define the observed Фe,C as apparent values that are included artifacts from methodological issues, such as bottle effects, which can be minimized by a relatively shorter incubation time (~2 h) [122]. Moreover, studies are needed to develop the incubation-free techniques, such as cellular NADP(H) measurement [123].” (**L680-685**)

122.	Milligan AJ, Halsey KH, Behrenfeld MJ. Advancing interpretations of ^14^C-uptake measurements in the context of phytoplankton physiology and ecology. J Plankton Res. 2015;37: 692–698. doi:10.1093/plankt/fbv051

123.	Jones BM, Halsey KH, Behrenfeld MJ. Novel incubation-free approaches to determine phytoplankton net primary productivity, growth, and biomass based on flow cytometry and quantification of ATP and NAD(H). Limnology and Oceanography: Methods. 2017;15: 928–938. doi:10.1002/lom3.10213

And, I am still concerned about the temporal offset between the in situ FRRf numbers and the in lab CO2 uptake numbers, which would go some way towards addressing the inconsistency, I think.

The morning in situ FRRf numbers are likely before any induction of community level photoinhibition.

The afternoon in lab CO2 uptake numbers may be after the onset of community level photoinhibition, or possibly even mid-day limitation on available DIC.

But, as the authors point out, if morning FRRf ETR is exaggerated relative to afternoon CO2 uptake, this would push phi e, C up, not down.

>>> We agree with your comment. We modified the sentence to clarify the mechanisms of the diel variation of phytoplankton productivity as follows,

“Previous studies reported that the carbon fixation rate of natural communities is ~1.3 times higher in the morning than in the afternoon because photoinhibition and nutrients depletion suppress community productivity in the afternoon [34,109]. If we underestimated carbon fixation rates of the communities incubated in the growth chamber, the results might have overestimated Фe,C. However, Фe,C values were paradoxically smaller than theoretical values (4 mol e− mol C−1) during most of the observations (Fig.4), except on 28 August, 13 September … ”(L**563-567**)

Specific Comments

Abstract:

"The GPP values estimated by FRRf ( GPP f ) with the best Ф e,C model relative to 13 C ( GPP 13C ) varied in the range of 0.5–1.5."

This is possibly predictively useful, but mechanistically impossible.

It is worth mentioning that Ф e,C cannot, mechanistically, be less than 4, and is more likely 5 or above.

>>> We inserted the sentence in abstract as follows,

“~carbon fixation (Ф_e,C_), which is mechanistically 4 mol e^−^ mol C^−1^ or above, can vary depending on multiple factors.” (L30)

"Thus, when cyanobacteria are dominant in a community, FO and FO′ could be underestimated (Campbell et al. 1998), "

Actually, when cyanobacteria are dominant it leads to an over-estimation of PSII derived FO, because phycobiliproteins contribute to measured F0.s

>>> Thank you for pointing this out. We corrected the sentences as suggested (L251-252).
